# A Novel Functional Role for *MMSET* in RNA Processing Based on the Link Between the REIIBP Isoform and Its Interaction with the SMN Complex

**DOI:** 10.1371/journal.pone.0099493

**Published:** 2014-06-12

**Authors:** Fabio Mirabella, Alexander Murison, Lauren I. Aronson, Christopher P. Wardell, Andrew J. Thompson, Sarah J. Hanrahan, Jacqueline H. L. Fok, Charlotte Pawlyn, Martin F. Kaiser, Brian A. Walker, Faith E. Davies, Gareth J. Morgan

**Affiliations:** 1 Centre for Myeloma Research, Division of Molecular Pathology, The Institute of Cancer Research, Sutton, United Kingdom; 2 Proteomics Core Facility, The Institute of Cancer Research, London, United Kingdom; CNRS UMR7275, France

## Abstract

The chromosomal translocation t(4;14) deregulates *MMSET* (*WHSC1*/*NSD2*) expression and is a poor prognostic factor in multiple myeloma (MM). *MMSET* encodes two major protein isoforms. We have characterized the role of the shorter isoform (REIIBP) in myeloma cells and identified a clear and novel interaction of REIIBP with members of the SMN (survival of motor neuron) complex that directly affects the assembly of the spliceosomal ribonucleic particles. Using RNA-seq we show that REIIBP influences the RNA splicing pattern of the cell. This new discovery provides novel insights into the understanding of MM pathology, and potential new leads for therapeutic targeting.

## Introduction

The t(4;14) chromosomal translocation, present in 10–15% of MM cases, is associated with a significantly worse prognosis compared to other genetic subgroups [1]. As a result of the translocation two oncogenes are overexpressed, *FGFR3* and *MMSET*. *FGFR3* over-expression has weak transforming properties and is eventually lost in 30% of patients [2], [3]. These findings suggest a critical role for *MMSET* in promoting t(4;14) myeloma which could be therapeutically targeted.

**Figure 1:**
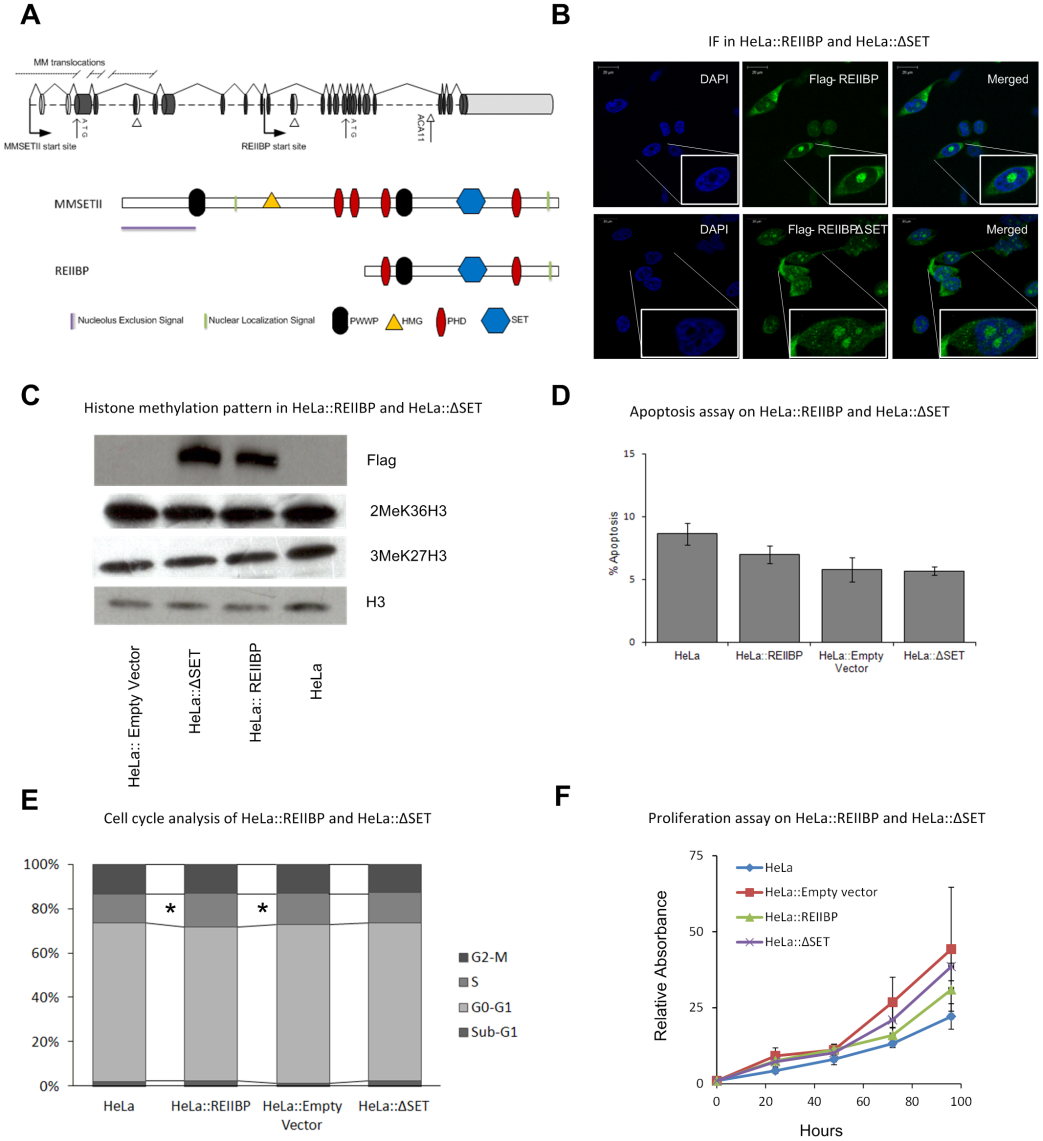
Overexpression of REIIBP in HeLa cells does not lead to changes in the pattern of histone methylation and lacks a clear cellular phenotype. A) Top panel shows the structure of WHSC1 gene. The position of the different translocation events in MM is shown by slashed lines. Grey and black ovals indicate non-coding and coding exons respectively, dotted straight line between exons represent the introns. Splicing event for the canonical longest MMSET isoform is shown by joining lines between exons. Exons 4a and 11, shown by white triangles, are used only for alternative splicing and are not present in MMSET II and REIIBP isoforms. Transcription start sites and first translated codons (ATG) for MMSET II and REIIBP are indicated respectively by bent black arrows and open arrows. Genomic position for ACA11 is shown by a white arrow. Bottom panel shows the protein structure of the two major isoforms of the whcs1 gene. The major domains of the proteins are, PWWP (Pro-Trp-Trp-Pro motif) a chromatin associated domains, HMG high mobility group, PHD plant homeo domain, SET set domain with pre and post motifs. The position of the nucleolus exclusion signal and nucleus localization signal sequences are based on published observations [15] and prediction analysis software [58]. B) IF and confocal analysis on HeLa::REIIBP and HeLA::ΔSET. REIIBP-Flag (green) localizes in the cytoplasm and nucleolus in HeLa cells overexpressing REIIBP-Flag. Single nucleolus zoomed in as inset in each frame. C) Western blot analysis on nuclear extracts. The antibody used is indicated on the left. D) Percentage of Apoptotic cells in the shown line. Error bars come from the SEM (standard error of the mean) of 3 different analysis. E) Percentage of cells at different stages of the cell cycle. Each data is the average of three analysis. * p value <0.05. F) WST-1 proliferation assay for the indicated cells. Error bars are the SEM of 5 different analysis. C–F) The control is the empty lentivector transduced into HeLa. See also Figure S1.

*MMSET* is a member of the NSD family of SET domain containing oncogenes known to be important in acute leukaemia and other malignancies [4]. It encodes multiple isoforms the most common being MMSET II, with another major isoform being REIIBP (Figure 1 A). In addition to having a variety of transcripts, recent work has found a small nucleolar RNA (snoRNA), *ACA11*, encoded within intron 18–19 of the *MMSET* gene (Figure 1 A), which is also highly expressed [5]–[7]. Its function is uncertain but it has been shown to bind to proteins involved in post-splicing intron complexes.

Pathological deregulation of *MMSET* deregulates genes involved in cell cycle, apoptosis and adhesion and there is strong evidence that MMSET II exerts its transforming properties through the histone Lysine methyltransferase activity of the SET domain [8], [9]. MMSET II has been shown to mediate histone methylation of H3K4, H3K27, H3K36 and H4K20 [9]–[13] but the consensus view is that, in terms of chromatin organisation, its predominant role is in dimethylation of H3K36 [14].

Interestingly, the study of translocation breakpoints from patient samples has shown that the breakpoints on chromosome 4 cluster in a limited number of regions in the 5′ region of *MMSET*. Analysis of transcripts derived from these different breakpoints is consistent with the expression of different truncated protein variants depending upon the site of the break. However, the only consistently expressed variant across all samples is REIIBP, suggesting it may be of particular importance (Figure 1 A). In contrast to MMSET II, which is confined only to the nucleus, REIIBP is located in both the cytoplasm and the nucleus [8], [15], suggesting *MMSET* has additional functional roles, which have yet to be described.

The SMN complex is a multi-subunit protein complex, which catalyses the assembly of snRNPs (small nuclear ribonucleic particles) that generate the RNA splicing machinery [16]–[19]. It is composed of the SMN protein together with the GEMIN 2–8 and UNRIP proteins [20], [21]. A point mutation (5% of cases) or a deletion (95%) of the *SMN1* gene, which encodes the SMN protein, results in the inherited neurodegenerative disease spinal muscular atrophy. The cytoplasmic SMN complex catalyses the binding of a hetero-heptameric ring of proteins, termed the Sm proteins, to the U-rich site in the splicing snRNAs (small nuclear RNA) [17]–[19]. After coupling to Sm proteins, the snRNA is hypermethylated at the 5′ end and then, while still attached to the SMN complex, is transported to the nucleus. In the nucleus these proteins localize to Cajal bodies where the snRNPs undergo maturation before being released from the SMN complex and exert their functional role as components of the spliceosome machinery.

Attempts to understand the functional role of REIIBP have been hindered by the lack of antibodies able to distinguish different *MMSET* isoforms together with the difficulty of knocking down REIIBP alone. In this work we have evaluated the role of REIIBP using a multi-tagged REIIBP construct artificially transduced into a MM cell line background. This work demonstrates that REIIBP binds to the SMN complex and that REIIBP methyl transferase activity alters snRNPs cellular abundance, affecting RNA splicing. This novel cytoplasmic mechanism for *MMSET* opens new perspectives in understanding the pathogenesis of t(4;14) myeloma.

## Materials and Methods

### Cloning

The cDNA for the MMSET isoform REIIBP was cloned in frame with the C-terminal double tags 6×HIS-3×FLAG and, through an auto-catalytic E2A peptide, linked to green fluorescence protein (GFP). The ΔSET constructs were generated by point mutating Asparagine 359, present in the SET domain, to Alanine (Agilent Technologies). The construct was cloned into a lentivirus system (pLVTHM or pRRLSIN) substituting the original GFP gene.

### Cell lines and viral transduction

H929 and HeLa cell lines were obtained from ATCC, and were grown in RPMI1640 containing GlutaMax (Life Technology), and DMEM (Life Technology) respectively, both media were supplemented with 10% heat-inactivated fetal calf serum (PAA Laboratories). Cells were cultured at 37°C in a humidified gas chamber with 95% air and 5% carbon dioxide. All cell lines used were mycoplasma-free as confirmed by PCR. Cell identities were confirmed by short tandem repeats analysis. Proliferation assays, western blotting, apoptosis assays and cell cycle analysis were conducted as described previously [22]–[24]. The lentivirus constructs were transduced into a myeloma cell line as described previously [25], [26]. See also Experimental procedures in Text S1.

### Tandem affinity purification of REIIBP partners

Around 5×10^8^ H929 cells were transduced with tagged REIIBP (H929::REIIBP) or H929 untouched used as control, and then harvested in 50 mM NaH_2_PO_4_ pH 7.4; 50 mM NaCl; 0.05% Tween; protease inhibitor (ROCHE). Cells were lysed by 3 cycles of freeze/thawing on dry ice and passed through a 10 gauge needle. Lysates were cleared by passing through 0.45 µm filter, spinning at 13000 rpm for 10 minutes and finally by incubating with 1 mL Agarose resin (Thermo Scientific). Equal amounts of protein from sample and control cleared lysates were loaded into chromatography columns filled with M2 anti-FLAG resin (SIGMA) and agitated at 4°C for 1 hour. Columns were washed with 50 mM NaH_2_PO_4_ pH 7.4; 300 mM NaCl; 0.05% Tween; protease inhibitor. Immuno complexes were eluted with 100 µg/ml 3×FLAG peptide (SIGMA). Eluates from the anti-Flag column were incubated for 1 hour in NiNTA agarose resin (Thermo Scientific) and washed with 50 mM NaH2PO4 pH 7.4; 300 mM NaCl; 20 mM Imidazole; 0.05% Tween; protease inhibitor. NiNTA columns were eluted with 250 mM Imidazole.

### Sample digestion and mass spectrometry analysis

Reagents were purchased from Sigma-Aldrich (Poole, UK) unless otherwise stated. 1D SDS-PAGE gel lanes corresponding to double affinity purification from H929::REIIBP and H929 were excised equivalently and in their entirety as 9 sections each. The gel sections were digested in-gel with trypsin and analysed by liquid chromatography-mass spectrometry as previously described [27] with the following adjustments: peptides were resolved on a 75 µm I.D. 15 cm C18 packed emitter column (3 µm particle size; NIKKYO TECHNOS CO., LTD, Japan) over 30 min, and ionised by electrospray ionisation using 1.7 kV applied immediately pre-column to the packed emitter via a microtee built into the nanospray source. Sample was infused into an LTQ Velos Orbitrap mass spectrometer directly from the end of the tapered tip silica column (6–8 µm tapered tip). Raw MS/MS data were compiled into peaklists, and interrogated against a SwissProt 2011_01 *homo sapiens* subset database (20,282 sequences), customised to include the REIIBP construct sequence, using Mascot v2.2 (www.matrixscience.com). A precursor ion tolerance of 5 ppm and fragment ion tolerance of 0.25 Da was applied. Relative protein enrichment was inferred from the spectral counts observed for each protein between the control and H929::REIIBP samples [28]. For the purpose of assessing enrichment, the protein acceptance probability was decreased to 80%. Spectra at this threshold were confirmed to be of good quality by visual inspection, and no false discovery hits against a reversed sequence decoy database were reported at this confidence level. See also Experimental procedures in Text S1.

### Co-immunoprecipitation

Myeloma cell lines were harvested in RSB buffer (100 mM NaCl; 2,5 mM MgCl_2_; 0.1% NP40; 10 mM Hepes pH 7.4; Protease inhibitor), and lysed by passing through a 19 gauge needle. Lysate was cleared by centrifugation and through filtering with a 0.45 µm strainer. Equal amount of protein lysate was immunoprecipitated with 2 µg of anti GEMIN5, SMN and normal mouse IgG (Millipore) for 2 hours at 4C. Immune complexes were harvested using Protein G conjugated to magnetic beads (Thermo Scientific) using manual recommendations.

### snRNPs quantification

HeLa cells were detached by trypsin, washed and lysed as described above. 800 ng of protein lysate were immunoprecipitated with 2 ug of anti SmB/B′/N (Santa Cruz). Equal amounts of input lysate were harvested and RNA extracted. Immune complexes were purified and RNA extracted and reverse transcribed as described above. cDNA was analysed by quantitative RTPCR (qRT-PCR) using syber green and a relative standard curve method. Data was normalized against mRNA actin values present in the inputs.

### RNA seq

Total RNA was purified using the Trizol (Life Technology) method. Total RNA from 3 replicates of HeLa cell line transduced with cDNA coding for REIIBP and 3 replicates of the control cell line (HeLa) was extracted. These 6 libraries were pulled down and split between 2 lanes of an Illumina HiSeq by Oxford gene technology. Raw fastq files were aligned using Tophat 2.0.8 (http://tophat.cbcb.umd.edu/) with default parameters, and Cufflinks 2.1.1 (http://cufflinks.cbcb.umd.edu/) was used to estimate transcript abundance and calculate differential expression between REIIBP and control samples. The results were visualized using cummerbund 2.0.0 (http://compbio.mit.edu/cummeRbund/). See also Experimental procedures in Text S1.

### Immuno fluorescence and confocal microscopy

0.1×10^6^ HeLa cells were seeded in a 13 mm cover slip for 5 hours and then fixed with 4% formaldehyde (Thermo Scientific) for 20 minutes at room temperature. Suspension cells were fixed in 4% formaldehyde and then cytospun at 300 rpm for 5 min onto microscope slides. Fixed cells were permeabilised in PBST buffer (1%BSA; 0.1%Triton-X in PBS) for 30 minutes in a humidified chamber, then blocked with 5% goat serum (SIGMA) in PBST. Permeabilised cells were stained for 1 hour at room temperature with one or two of the following primary antibodies: FLAG (SIGMA; Thermo Scientific); coilin (Santa Cruz); SMN, GEMIN5 (Millipore); Fibrillarin (Cell Signalling); REIIBP [8] and then for 1 hour with secondary Alexa Fluor (448, 566 or 633) antibodies. Stained cells were mounted using Vectashield media with DAPI. Confocal analyses were conducted on a Zeiss LSM700 equipped with inverted Axio Observer.Z1 and AxioCam. The lenses used were Plan-Apochromat 40×/1.3 and 63×/1.40. Immersion oil used is Immersol 518F (ZEISS). Images were acquired using ZEN 2009 (ZEISS) software and analysed using ZEN 2009 (ZEISS) or Image J softwares.

## Results

### Overexpression of REIIBP in HeLa cells does not lead to changes in the pattern of histone methylation and lacks a clear cellular phenotype

In order to study REIIBP physiology we virally transduced HeLa cells with an REIIBP construct (HeLa::REIIBP) or an REIIBP construct with an inactivating mutation in the SET domain (HeLa::ΔSET). Both constructs carried HIS-FLAG tags (Figure S1A). HeLa cells were chosen as the initial model for our study because MMSET is present at very low levels (Figure S1B) providing a background in which to study the role of REIIBP without interference from other MMSET isoforms. In order to confirm REIIBP expression and cellular localization, HeLa::REIIBP clones were FACS sorted, and then underwent immunofluorescent staining (IF) with confocal microscopy imaging and western blotting. Cellular fractionation and western blot assays on transduced HeLa lines confirmed previous data in MM cells [8] where REIIBP was found both in the nucleus and the cytoplasm (Figure S1B). IF and confocal imaging of HeLa lines showed that while REIIBP staining is widely dispersed throughout the cytoplasm, its nuclear signal was confined to the nucleolus (Figure 1B), a finding confirmed by co-localisation with Fibrillarin, a nucleolus marker (Figure S1C). The nucleolar localization of REIIBP had previously been suggested in other cellular systems where REIIBP was artificially introduced [15], [29]. We confirmed the same pattern of REIIBP localization, in the cytoplasm and in the nucleolus, in myeloma cells by IF staining H929 t(4;14) cells using an antibody with higher affinity for REIIBP than MMSET II [8] (Figure S1D). In a minor proportion of cells, REIIBP was only seen in the cytoplasm, without a detectable signal in the nucleolus.

It has previously been reported that REIIBP can dimethylate H3K36 in isolated nucleosomes [14], but a similar construct *in vivo* lacked this effect [9], [30]. Similarly to these reports, we found that our system did not produce any change in the global histone methylation levels of H3K36me2 or H3K27me3 as would have been expected if MMSET II had been used in such an experiment (Figure 1 C). This lack of an effect on the methylation of nuclear histones possibly reflects the fact that REIIBP and MMSET II have different localization within the nucleus, with REIIBP being found only in the nucleolus consistent with having a distinct role from MMSET II.

In order to characterize the role of REIIBP, we carried out a series of biological assays on transduced cells. We could not detect any difference in the number of cells in apoptosis or proliferation rate as determined by a WST-1 assay in the transduced cells. We found a small but significant increase in the number of HeLa::REIIBP cells in S phase of the cell cycle (Figure 1 D–F).

The lack of a distinct cellular phenotype for HeLa::REIIBP cells suggests that the biological relevance of REIIBP may be limited to specific cellular backgrounds, such as plasma cells. We sought to investigate this further by identifying REIIBP binding partners present in myeloma cells.

### REIIBP interacts with the SMN complex in t(4;14) myeloma cells

We characterized REIIBP interacting partners by tandem affinity purification followed by mass spectrometry. In these experiments an REIIBP construct carrying HIS/FLAG tags was transduced into the t(4;14) positive H929 myeloma line (H929::REIIBP). In order to avoid artefacts introduced by differences in REIIBP expression, we selected clones expressing tagged REIIBP at lower levels than the endogenous REIIBP by FACS.

**Figure 2:**
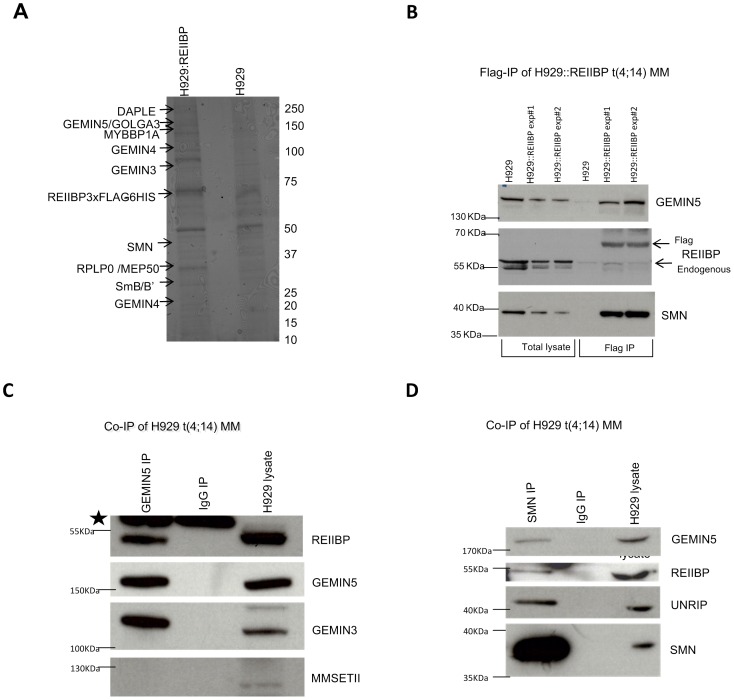
REIIBP interacts with the SMN complex in myeloma t(4;14) cells. A) Identification of REIIBP binding partners. REIIBP protein partners were pulled down by a tandem affinity purification approach. To isolate a protein fraction with a low level of background contaminants, the lysates from H929::REIIBP and H929 (control sample) were passed through two different chromatography columns, an anti-Flag resin and Nickel resin. The purified populations were run on SDS acrylamide gel and stained with Coomassie blue. Discrete bands were cut from the gel and analysed by mass spectrometry. The number on the right indicates the position of the respective molecular weights in KDa. The positions for the proteins identified by Mass spectrometry are shown by arrows. B) Western blot analysis on Flag IP fractions. The names of the respective antibodies used for staining are shown. Molecular weight is shown. C–D) Endogenous REIIBP interacts with the SMN complex in myeloma cells. Co-IPs of GEMIN5 (C) and SMN (D) in t(4;14) untransduced myeloma cells. GEMIN5 and SMN were immuno precipitated from H929 cell lysates using specific antibodies. An IP with normal mouse IgG antibody was used as control. The IPs were resolved in a SDS acrylamide gel and blotted for the indicated antibodies. The black star shows the position for the heavy chains of the antibodies used in the Co-IP. Please note MMSET II in H929 cells has a shorter molecular weight than canonical 150 KDa.

Mass spectrometry analysis on these cells (Figure 2 A) identified over 200 proteins, which were prioritised by fold enrichment using spectral counting. Proteins exhibiting at least 10 fold enrichment compared to control (Table 1) included a number of different RNA processing factors. In particular, we found evidence of a strong association with 4 members of the SMN complex (GEMIN3, 4, 5 and SMN). In addition, all the remaining members of the SMN complex (GEMIN2, 6–8 and UNRIP) were also identified, as well as a number of ancillary proteins of the SMN complex, including members of the RNA splicing machinery (SF3B3, U2SURP), although these were detected at lower levels.

**Table 1:** List of proteins identified as co-enriched with REIIBP by mass spectrometry.

	Identified Proteins	Gene ID	Spectral Counts	Unique Peptides	Sequence Coverage	Molecular Weight
	REIIBP Flag HIS construct		219	44	58%	101 kDa
**★**	Component of gems 4	*GEMIN4*	97	44	49%	120 kDa
**★**	Gem-associated protein 5	*GEMIN5*	69	38	31%	169 kDa
**★**	Probable ATP-dependent RNA helicase DDX20	*DDX20*	58	25	43%	92 kDa
	Golgin subfamily A member 3	*GOLGA3*	45	29	21%	167 kDa
	Myb-binding protein 1A	*MYBBP1A*	35	26	25%	149 kDa
	Plectin	*PLEC*	32	31	7%	532 kDa
	Protein Daple	*CCDC88C*	28	20	11%	228 kDa
	60S ribosomal protein L6	*RPL6*	27	6	24%	33 kDa
	RNA-binding protein 10	*RBM10*	20	16	21%	104 kDa
	Phosphoribosyl pyrophosphate synthase-associated protein 2	*PRPSAP2*	19	12	38%	41 kDa
	Thyroid hormone receptor-associated protein 3	*THRAP3*	17	13	15%	109 kDa
	60S acidic ribosomal protein P0	*RPLP0*	17	12	49%	34 kDa
	Tyrosine-protein phosphatase non-receptor type 13	*PTPN13*	15	14	6%	277 kDa
**★**	Survival motor neuron protein	*SMN1*	15	10	43%	32 kDa
	Kinesin-like protein KIF11	*KIF11*	14	11	11%	119 kDa
**★**	Methylosome protein 50	*WDR77*	14	6	19%	37 kDa
	Probable E3 ubiquitin-protein ligase MYCBP2	*MYCBP2*	13	11	3%	510 kDa
	Microtubule-associated protein 1B	*MAP1B*	12	12	6%	271 kDa
	Superkiller viralicidic activity 2-like 2	*SKIV2L2*	10	9	10%	118 kDa

Proteins are ordered relative to the number of mass spectra assigned to peptide sequences unique to each protein. Only proteins with at least 10 fold enrichment in spectral counts relative to control are shown. Sequence coverage denotes the percentage of total amino acid sequence covered by the peptides detected. Asterisks denote known members of the SMN complex: GEMIN4, GEMIN5, GEMIN3 (ddx20), SMN and MEP50 (wdr77).

In order to validate the REIIBP/SMN interaction, we carried out western blotting on H929 and H929::REIIBP following anti-FLAG pull-down (Figure 2 B) using antibodies against different members of the SMN complex. We identified specific bands derived from members of the SMN complex only in the H929::REIIBP lanes, confirming the mass spectrometry result, that REIIBP is bound to the SMN complex.

In order to demonstrate that this interaction was not mediated via binding through the FLAG or the HIS tags, and at the same time to rule out the possibility that the interaction was due to REIIBP overexpression, we investigated the interaction in an untransduced H929 line. GEMIN5 co-immuno-precipitation (Co-IP) pulled down GEMIN3, a member of the SMN complex (Figure 2 C). Importantly, we also found a specific signal for endogenous REIIBP confirming its interaction with the SMN complex. However, GEMIN5 was not able to Co-IP the MMSET II isoform. Co-IP of the SMN protein showed a similar result (Figure 2 D), confirming that endogenous REIIBP is bound to the whole SMN complex, and not only to certain members.

To support the Co-IP data we used confocal microscopy to analyse H929 cells immuno-stained with SMN and REIIBP antibodies. Analysis of several optic fields showed that REIIBP co-localized with SMN in the cytoplasm (Figure S2).

Collectively, these results confirmed the novel interaction of REIIBP with the SMN complex.

### REIIBP alters cellular snRNPs levels

Previous reports have demonstrated that knock down of the SMN protein causes a reduction in mature snRNPs and changes the RNA splicing patterns [31], [32]. In order to verify whether REIIBP could influence the stability of the SMN complex and hence alter the production of splicing RNPs we made further use of our HeLa::REIIBP line.

To confirm here the co-localization between the SMN complex and REIIBP we double stained HeLa::REIIBP and HeLa cells with antibodies against FLAG, SMN and the GEMIN5 epitopes (Figure S3). Consistent with the data above we show that the SMN signal is present only in the cytoplasm and in the nuclear Cajal bodies, while the FLAG signal, derived from REIIBP, is detected in the nucleolus and cytoplasm. GEMIN5, as previously reported [33], was not only localized in the Cajal bodies, but was present also as a dispersed signal throughout the nucleoplasm. However, both SMN and GEMIN5 showed a signal consistent with extensive cytoplasmic co-localisation with REIIBP. Analyzing the pattern of the co-localization signals for GEMIN5 and REIIBP (bottom Figure S3 B) we found a high degree of co-linearity in the cytoplasm, which was lower in the nucleus. We also looked specifically for the presence of REIIBP with the SMN complex in the nuclear Cajal body but could find no evidence for co-localization at this site. Furthermore, we did not observe differences in either the number or distribution of the Cajal bodies within the cell between the native HeLa and the transduced cells (Data not shown).

IF and confocal analysis of HeLa::ΔSET also showed co-localization with GEMIN5 (Figure S4) suggesting that the methyltransferase activity of the SET domain is not responsible for mediating binding to the SMN complex.

**Figure 3:**
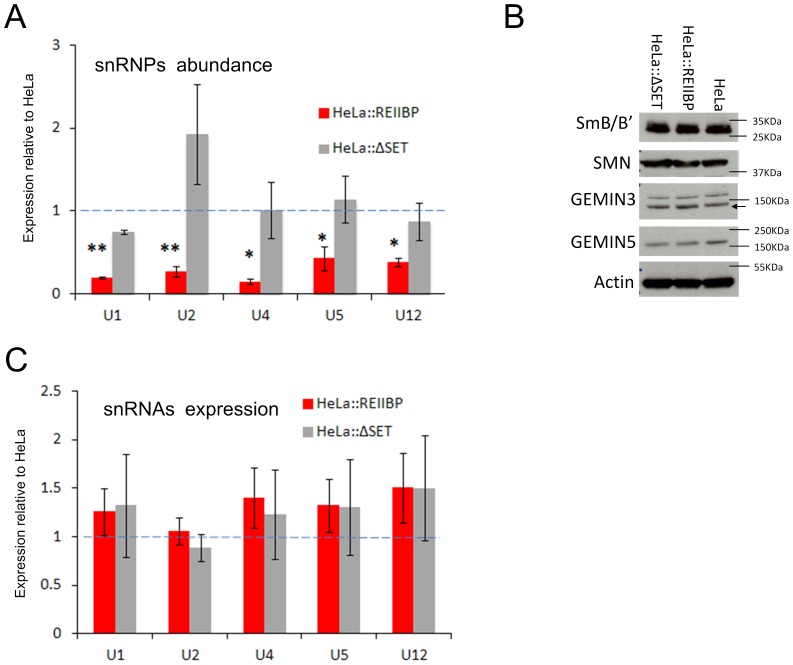
REIIBP SET domain controls spliceosomal snRNPs abundance. A) Cellular snRNPs abundance. snRNPs were immunoprecipitated with anti SmB/B′ antibody, snRNAs were purified, reverse transcribed and quantified by qPCR, (compared to the level of βactin mRNA present in the input). Each analysis is an average of three independent IPs. B) SMN complex components and SmB/B′ protein levels. Total cell lysates were immunoblotted against the relative antibodies. The black arrow indicates GEMIN3 specific bands. c) snRNAs expression analysis. qRT-PCR of total RNA. Each value is an average of three independent analysis. Figures A and C are expressed relative to control (HeLa cells, dotted line). The error bars are the SEM. ** p value <0.006, * p value <0.05.

We hypothesised that the REIIBP/SMN interaction could influence the formation of new splicing snRNPs. In order to address this question we immunoprecipitated cellular splicing snRNPs in HeLa::REIIBP, HeLa::ΔSET and HeLa lines, using an antibody against the Sm protein SmB/B′, we extracted total RNA from the immunoprecipitates and compared the level of snRNAs (U1, U2, U4, U5 and U12) by qRT-PCR (Figure 3 A). Results demonstrate that REIIBP overexpression causes a major reduction in the abundance of snRNPs in the cell, in both the major (U4) and minor (U12) splicing components. This observation is in contrast to what is seen in SMN protein knockdown [31] where it is the U12 minor splicing components that are predominantly affected. This decrease was not observed in HeLa::ΔSET, consistent with the methyltransferase activity of REIIBP being responsible for this effect. In order to exclude the possibility that snRNPs variation is not due to the loss of the SMN complex or to a decrease in the level of SmB/B′ proteins, immunoblot analysis of SmB/B′ and SMN complex components were carried out, showing no difference in the three lines (Figure 3 B). In some cases REIIBP has been reported to repress transcription [10]. To refute the possibility that REIIBP could modify the snRNPs abundance by altering the gene expression of snRNAs, we measured the expression level of snRNAs in the transduced HeLa lines, and found no differences (Figure 3 C).

### REIIBP induces changes in RNA splicing patterns

**Figure 4:**
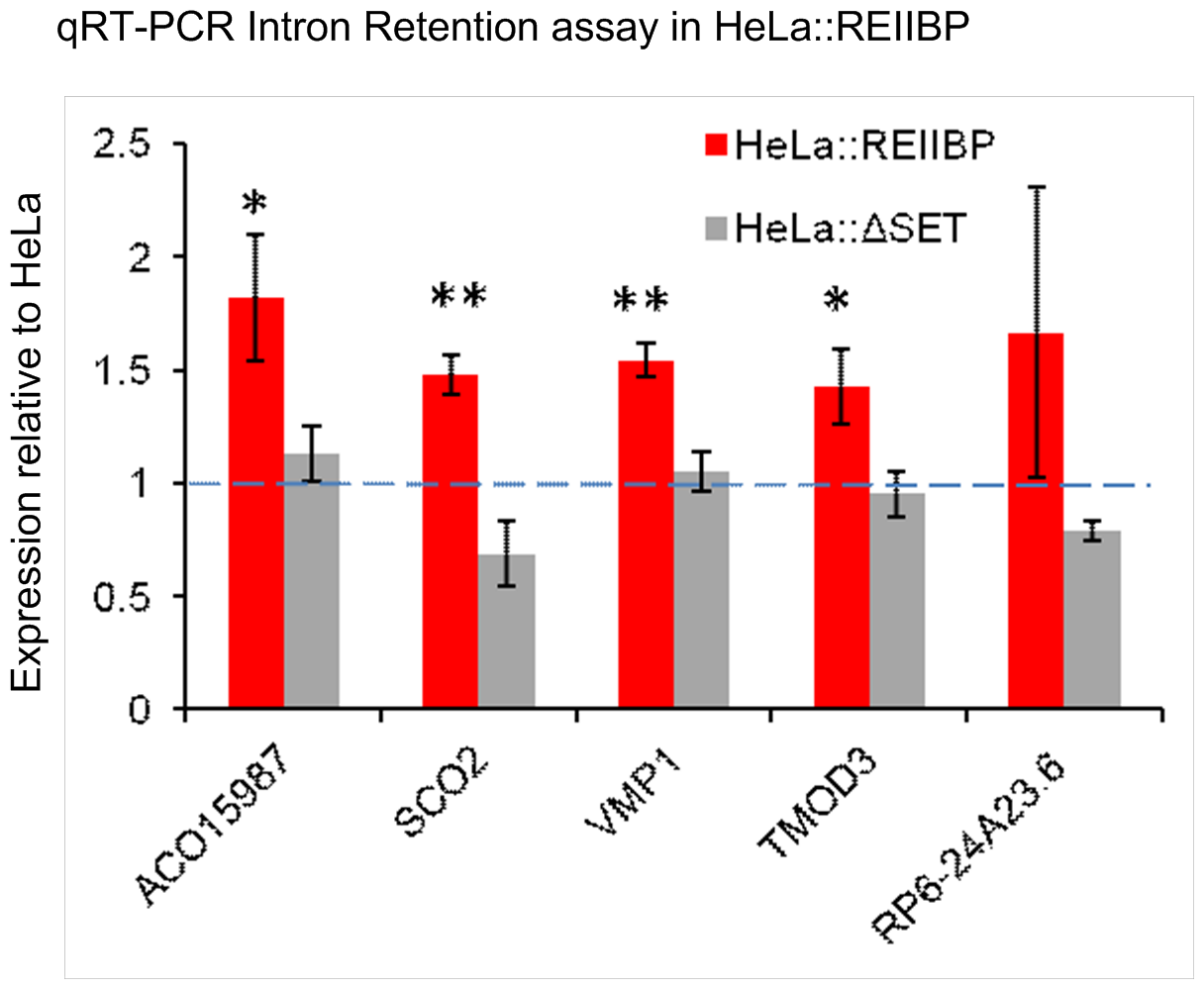
Intron retention assay. Intron retention qRT-PCR results for a subset of genes present in Table 2. Intron values for the indicated genes are normalized against the nearest exons and the βactin mRNA values. Data are shown as relative to HeLa values (blue dotted line). Each analysis is an average of at least three independent experiments. The error bars shown are the SEM, ** p value <0.005, * p value <0.05.

snRNPs make up the bulk of the spliceosome machinery structure. In order to test if the decreased number of snRNPs in HeLa::REIIBP deregulates downstream RNA splicing patterns, we used RNAseq of the REIIBP transduced cells compared to control HeLa cells. We focused on changes in splicing patterns looking for gene specific alternative isoforms as well as for the presence of new transcripts generated by exon skipping or intron retention. We found a wider variety of transfrags showing intron retention in HeLa::REIIBP, with a statistically significant difference between the proportion of unique transfrags corresponding to annotated exons and at least 10 bp of intron identified between the HeLa samples and HeLa::REIIBP samples (p = 0.027, 2 tailed unpaired t-test). A list of the genes most affected by intron retention is shown in Table 2. We confirmed these results using qRT-PCR to analyse the amount of retained intron in the mRNA of a subset of the genes shown in Table 2 (Figure 4). HeLa::REIIBP had an increased amount of intronic mRNA compared to control cells. Interestingly intronic values in ΔSET::REIIBP were equivalent to HeLa control, confirming the role of the SET domain in controlling splicing defects. Moreover, we found evidence of around 120 spliced isoform transcripts differentially expressed between the two conditions (Table S1). Overall these results show a clear difference in RNA splicing patterns associated with the overexpression of REIIBP.

**Table 2:** Shortlist of genes with intron retention identified by RNAseq analysis.

Gene ID	ENSEMBL ID	Average Difference	Chromosome	Start	End
*YY1AP1*	ENST00000436865	634.10	1	155648847	155649158
*HIST1H2BK*	ENST00000396891	334.40	6	27114160	27114662
*RCC1*	ENST00000373833	6.15	1	28832567	28843007
*RP11-939C17.2*	ENST00000531652	8.61	11	75283076	75285403
*HIST1H2BD*	ENST00000289316	122.06	6	26156402	26161402
*HIST1H4H*	ENST00000289352	66.68	6	26284668	26285817
*VMP1*	ENST00000262291	18.32	17	57915269	57920852
*AC015987.1*	ENST00000419211	24.30	7	135610856	135613214
*CTC-338M12.2*	ENST00000513771	10.43	5	180618535	180618759
*TMOD3*	ENST00000560549	12.79	15	52201456	52207126
*LRFN4*	ENST00000393952	−8.48	11	66624758	66625448
*AL589743.1*	ENST00000418499	22.99	14	19680924	19685836
*RP6-24A23.6*	ENST00000563887	20.92	X	107963182	107973037
*SAP30*	ENST00000504618	5.55	4	174291216	174292405
*METTL12*	ENST00000532971	16.80	11	62432628	62434777
*SCO2*	ENST00000543927	11.38	22	50962024	50963145

The Gene ID and Esembl ID are indicated. The average difference shows the average difference of intron exon transfrags between HeLa and HeLa::REIIBP. The chromosomal location for the transfrags is also indicated. See also Table S1–S4.

In addition to the altered splicing, REIIBP overexpression affects the expression of a number of genes. Gene ontology analysis of these genes using the DAVID bioinformatic tool [34], identified enhanced expression of genes involved in differentiation (Table S2). This finding was also confirmed by gene pathway analysis, where a strong correlation with synaptic vesicle trafficking and axon guidance pathways was found (Table S3). Among the developmental genes activated by REIIBP, 54 homeotic genes were overexpressed (Table S4). We also found a number of long non coding RNA genes deregulated. Of interest we report a massive increase in the expression of sno13 RNA and the Xist RNA. We also noticed an activation of ephrin genes.

### REIIBP controls snRNPs levels in t(4,14) myeloma cells

**Figure 5:**
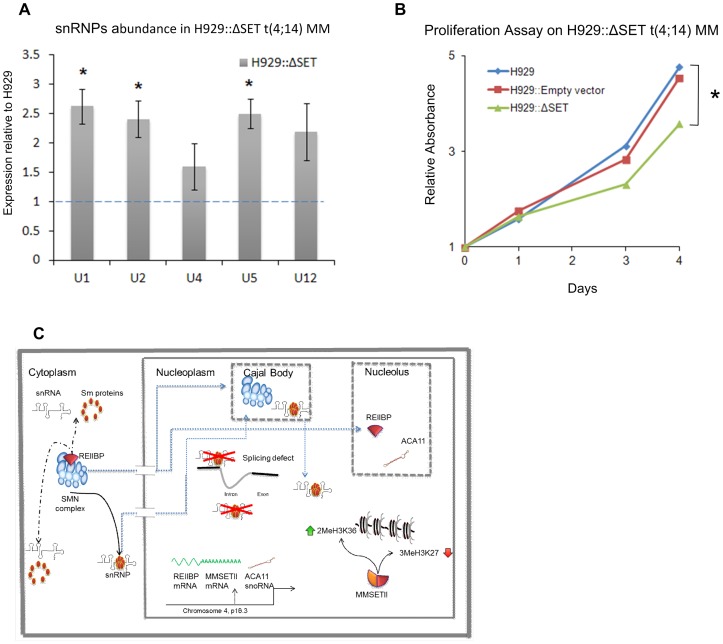
REIIBP controls snRNPs assembly and proliferation in myeloma cells. A) Cellular snRNPs abundance. snRNPs were immunoprecipitated and analysed as in Figure 3 A. Each analysis is an average of three independent IPs. Data are shown as relative to H929 values (blue dotted line). The error bars shown are the SEM. B) WST-1 proliferation assay for the indicated cells. Results represent the average of 3 independent assays. A–B) * p value <0.05. C) A schematic representation of the function and position of the MMSET gene and its products. MMSET II controls the di methylation on Lysine36 of Histone3 (2MeH3K36) and the tri methylation of Lysine 27 on histone3 (3MeH3K27) [9], [14]. Aca11 has been reported to be in the nucleolus [5], whilst the SMN complex has both cytoplasm and nucleus localization. The SMN complex is found in the nucleus at level of Cajal bodies or in the gem bodies. REIIBP and the SMN complex are likely to interact in the cytoplasm. REIIBP catalytic activity, possibly on the SMN complex, affects the number of spliceosome snRNPs particles causing splicing defects. The schematic representation of the SMN complex model is based on a previously proposed model [21]. The structures for REIIBP, ACA11, MMSET II and snRNPs are arbitrary.

We have demonstrated that the interaction of REIIBP with the SMN complex occurs in myeloma and possibly in HeLa cell lines as well. The technical difficulty of knocking down REIIBP alone in myeloma cells, without affecting MMSET II expression, prevented us from analysing the effect of the interaction between REIIBP and the SMN complex in t(4;14) MM cells. We, therefore, addressed this issue using a dominant negative approach. We overexpressed a mutant SET domain REIIBP construct in H929 cells (H929::ΔSET), this out competed endogenous REIIBP for gaining access to its natural methylation targets and therefore counteracting its function. Analysis of splicing snRNPs levels in H929::ΔSET showed a significant increase compared to H929 (Figure 5 A). This observation confirms REIIBP's role as a regulator of the spliceosome machinery in MM cells. Moreover, the steric competition of the mutant versus the endogenous REIIBP slowed the proliferation of MM cells (Figure 5 B), suggesting that REIIBP increases t(4;14) MM cell proliferation by altering the level of splicing snRNPs.

## Discussion

In this work we have characterized a novel role of REIIBP, the only protein product from the *MMSET* gene which is consistently deregulated by the t(4;14) translocation in myeloma. It has previously been shown that MMSET II enhances t(4;14) cell proliferation [8], [11], [14] and we have now demonstrated that REIIBP has a similar function but acting via an independent biochemical pathway (Figure 5 B–C). In this new role, the nucleo-cytoplasmic REIIBP protein mediates RNA splicing patterns via its methyltransferase activity. This is a novel mechanism in myeloma, but it is consistent with pathogenic events affecting gene splicing in other cancers [35]–[39]. It is known in other systems that mutations in genes such as *U2AF1, SF3B1, U2AF2* cause miss-annealing of snRNP at the correct 3′ splice site, leading to intron retention or exon skipping. Interestingly, in the context of the current report, knockdown of these factors leads to specific alterations in splicing patterns for only a subset of genes rather than causing global effects [40]–[43]. In contrast to the mutational events affecting splicing in other cancers, we find that RNA splicing machinery defects in t(4;14) MM are caused by REIIBP overexpression which results in impairment of SMN complex activity (Figure 5 A–C).

Regulation of SMN complex activity has already been shown to be relevant during development and cellular differentiation [44]. Moreover, the maturation and abundance of specific snRNPs, especially U1 and U2 snRNPs, are thought to be fine-tune controllers of alternative splicing, premature mRNA termination, alternative isoform expression and intron retention [32], [45]–[48]. It is clear, therefore, that deregulation of such processes provide a credible pathogenic mechanism in carcinogenesis.

In this report focusing on the role of REIIBP, we show that as a result of its overexpression, there are significant changes in the pattern of RNA splice variants at a limited number of genes, mostly affected by intron retention rather than occurring as a result of alternative splicing. This is in agreement with observations in other systems where a reduction in the level of snRNPs does not necessarily correlate with shifts in the patterns of alternative splicing but rather can lead to an increase in aberrant splicing products. These changes are seen in a limited subset of genes [31], and are often associated with tissue specific variation [32], [49]. It is therefore possible that the REIIBP induced defects in RNA splicing we demonstrate in HeLa cells would be different in a plasma cell background. In this regard we found that whilst REIIBP can influence snRNPs formation in different cellular contexts (Figure 3 A and Figure 5 A), it specifically deregulates proliferation in MM cells (Figure 5 B).

Intron retention is an important mechanism both in normal tissue regulation and in disease states. It is associated with granulocyte differentiation [50], spinal muscular atrophy onset [31], and developmentally regulated intron retention can act as switch for gene expression [51]. We suggest that analogous to these processes, intron retention plays a role in myeloma pathogenesis. We postulate that intron retention could produce mRNAs with frame shift-induced stop codons, which would then be degraded by cytoplasmic nonsense mediated decay or by the nuclear exosome. This would explain why intron retention is seen in only a limited set of genes. This hypothesis is supported by the large number of genes we found downregulated by REIIBP in our RNAseq data.

In terms of the sub-cellular location, we show that REIIBP co-localizes with the SMN complex in the cytoplasm (Figure S2, Figure S3 and Figure 5 C). Within the nucleus REIIBP is found in the nucleolus (Figure 1B, Figure S1C–D). In contrast, the SMN complex is predominantly located in the cytoplasm and in the Cajal bodies where it exerts its role on snRNPs. As a consequence of REIIBP overexpression we saw no change in the structure or number of the Cajal bodies, ruling those out as being involved in the potential mechanism. There are therefore two possible mechanisms: either REIIBP modifies the SMN complex and once modified, the continued interaction is no longer necessary or alternatively the binding of REIIBP to the SMN complex in the cytoplasm is important not only to modify the SMN complex but also in mediating the nuclear transport of the two entities, which then dissociate. Experimental insights into these potential mechanisms come from the mass spectrometry and confocal data which show that REIIBP has a higher affinity for GEMIN3, 4 and 5 compared to other components of the SMN complex (Table 1). This trio of proteins interact strongly [52]: GEMIN5 is essential for binding and loading the snRNA [53], GEMIN3 acts as an RNA helicase giving access to the RNA, while the function of GEMIN4 is unknown. It is thus plausible that REIIBP catalytic activity could control the loading of snRNAs onto the complex, or alternatively it could diminish the number of functional SMN complex molecules. A different plausible mechanism is that REIIBP instead targets Sm proteins. Sm proteins, before being assembled into snRNP particles, are normally Arginine dimethylated. However, it has also been shown that Sm protein can be Lysine methylated [54]. It is therefore possible that Lysine methylation by REIIBP could compete with canonical Arginine dimethylation, causing a reduction in the availability of functional Sm proteins. In order to prove this theory, further studies must be carried out to determine the precise REIIBP methylation target sites.

Further insight into the potential pathologic relevance of REIIBP overexpression comes from its localization within the nucleolus. *ACA11*, encoded within intron 18–19 of the *MMSET* gene, has been suggested to modulate the control of oxidative stress and to downregulate ribosomal protein genes and is also found in the nucleolus [5]. It is possible that REIIBP and *ACA11* could work in the same pathway, as it would be expected for a snoRNA and its host gene [55]. This hypothesis is supported by the observation that both *ACA11* and REIIBP interact with the nucleolar MYBP1A protein [5] (Table 1). Interestingly, the SMN complex also seems to interact with MYBP1A [56].

In terms of defining the specific biological roles of different *MMSET* isoforms, we could not detect a change in histone methylation pattern when REIIBP was over-expressed. The MMSET II isoform is known to alter histone methylation patterns and the difference with REIIBP possibly reflects the fact that they have different nuclear localization and that REIIBP lacks the upstream PWWP and PHD domains which mediate DNA binding [30]. In contrast to REIIBP, MMSET II is dispersed throughout the nucleoplasm, where it has important roles in regulating H3K36me2 levels. Moreover, when MMSET II was knocked out in KMS11 myeloma cells, expression of REIIBP and *ACA11* were unaffected [6], [57] but the epigenome landscape was different compared to the parental cells [9], [14]. These data are consistent with MMSET II having an important role in histone modification whereas REIIBP and *ACA11* seem to be important in RNA processing.

The observation that REIIBP does not mediate histone methylation suggests that the different patterns of gene expression we demonstrate as a result of REIIBP overexpression are the result of a different mechanism. Insight into this mechanism comes from analysis of the patterns of deregulated genes. We show that REIIBP overexpression increases the expression level of a number of developmental related genes and homeotic genes. In terms of this potential mechanism, it is reported that SF3B1 and U2 snRNP, both of which are components of the splicesomal machinery, interact with polycomb proteins in a splicing independent mechanism that mediates the repression of homeotic genes [41]. Thus it is possible that the REIIBP interaction with the SMN complex could influence repression of polycomb regulated homeotic genes by altering the equilibrium of U2 snRNPs. This could also explain the activation of non homeotic genes, like Xist, which are repressed by the polycomb complex.

Overall we describe a novel role for the REIIBP isoform of *MMSET* in modulating the function of the RNA splicing machinery via its interaction with the SMN complex. This could have an impact on downstream cellular functions such as homeotic gene expression and an important role in the pathogenesis of myeloma. We demonstrate that the SET domain is critical for the role of *MMSET* in RNA processing as well as its previously recognised role in modulating histone methylation patterns. *MMSET* overexpression is an important early event in myeloma that has multiple downstream effects and is associated with poor outcome. This could potentially be reversed by inhibiting its methyltransferase activity via the SET domain. Blocking the interaction between the SMN complex and REIIBP could also be investigated as a possible therapeutic target.

## Supporting Information

Figure S1 **Characterization of HeLa cells transduced with REIIBP constructs.** A) Schematic Protein structure of the transduced tagged REIIBP. B) Transduced HeLa lines correctly express REIIBP. The label “REIIBP” represents the HeLa cell line transduced with REIIBP double tagged with 6×HIS-3×FLAG. “ΔSET” is the HeLa cell line transduced with the same construct but with an inactivating mutation in the catalytic SET domain. Cytoplasm and nuclear fractions were isolated and analysed by western blot, using an antibody which recognizes both MMSET II and REIIBP isoforms or an antibody which recognizes Histone 3 (H3). KMS11 is a myeloma cell line used as positive control. The position for each of the respective protein species is indicated. C) IF and confocal analysis. REIIBP-Flag (green) co-localizes with the nucleolus marker Fibrillarin (red) in HeLa cells overexpressing REIIBP-Flag. The merged image includes DAPI (blue) staining. White arrows point to the same cellular region in all three frames. Single cell zoomed in as inset in each frame. Colocalization profile of Fibrillarin and Flag-REIIBP is show under the “merged” frame. The fluorescence profile is generated from the area covered by the red arrow in the merged picture. The blue plot represents the intensity of the DAPI stain; the green plot, the intensity of FLAG and the red the intensity of Fibrillarin. D) IF and confocal analysis on H929 t(4;14) MM cells. REIIBP (red) localizes in the cytoplasm and in the nucleolus. Single nucleolus zoomed in as inset in each frame.
(TIF)

Figure S2 **REIIBP co-localizes with the SMN complex in myeloma t(4;14) cells.** Immuno fluorescence and confocal analysis on H929 cells. A) REIIBP (green) co-localizes with SMN (red) in the cytoplasm. B) SMN (red) colocalizes with Coilin (green) at level of cajal bodies in the nucleus. The merged images include DAPI (blue) staining and white arrows show the nuclear position for the cajal bodies. Of note the antibody against REIIBP also recognizes the MMSET II isoform.
(TIF)

Figure S3 **REIIBP-Flag co-localizes with SMN and GEMIN5 in HeLa cells overexpressing REIIBP-Flag.** Immuno fluorescence and confocal analysis of HeLa::REIIBP-FLAG cells. A) Colocalization of FLAG (red) with SMN (green). Open arrows show the colocalization signal from the cytoplasm. Closed arrows indicate the nuclear position of Cajal bodies. B) Colocalization of FLAG (green) with GEMIN5 (red). Co-localization profile of GEMIN5 and REIIBP is shown at the bottom. The fluorescence profile is generated from the area covered by the red arrow in the merged picture. The blue plot represents the intensity of the DAPI stain; the green plot, the intensity of FLAG, the red the intensity of GEMIN5. C) SMN (red) co-localizes with Coilin (green) at level of cajal bodies in the nucleus. Merged images in A), B) and C) include DAPI (blue) staining.
(TIF)

Figure S4 **REIIBP-Flag with an inactivating mutation in the SET domain co-localizes with GEMIN5 in HeLa::ΔSET.** Immuno fluorescence and confocal analysis of HeLa::ΔSET cells. Colocalization of REIIBPΔSET-FLAG (green) with GEMIN5 (red). Merged image includes DAPI (blue) staining. Arrows show the colocalization signal from the cytoplasm.
(TIF)

Table S1 **Transcript isoforms alternatively spliced between HeLa and HeLa::REIIBP.** RNASeq result showing a list of differently expressed transcripts within genes where the overall expression was unchanged.
(DOCX)

Table S2 **Gene ontology analysis of genes overexpressed in HeLa::REIIBP.** List copiled using DAVID bionformatic resources (http://david.abcc.ncifcrf.gov/). Fold enrichment measures the magnitude of enrichment compared to the human genome. Fold enrichment >1.5 was considered as interesting. The percentage is the total number of genes involved in given term divided by the total number of input gene. P-value examine the significance of gene-term enrichment. P value <0.05 was considered significant.
(DOCX)

Table S3 **Gene pathway analysis of genes overexpressed in HeLa::REIIBP cells.** KEGG_PATHWAY analysis of genes overexpressed in HeLa::REIIBP cells. The list was copiled using DAVID bionformatic resources (http://david.abcc.ncifcrf.gov/). Fold enrichment measures the magnitude of enrichment compare to human genome. Fold enrichment >1.5 was considered as interesting. The percentage is the total number of genes involved in a given term divided by the total number of input genes. P-values examine the significance of gene-term enrichment. P value <0.05 was considered significant. PANTHER and REACTOME pathway analysis gave similar results.
(DOCX)

Table S4 **Polycomb regulated genes deregulated in HeLa::REIIBP cells.** RNASeq result showing differently expressed transcripts which are known to be silenced by the Polycomb complex.
(DOCX)

Text S1 **Supplemental Experimental Procedures.**
(DOC)
