# Supplementary figures and images for: A Novel Functional Role for MMSET in RNA Processing Based on the Link Between the REIIBP Isoform and Its Interaction with the SMN Complex
Source: PLoS One. 2014 Jun 12;9(6):e99493. doi: 10.1371/journal.pone.0099493 (PMC4055699; doi:10.1371/journal.pone.0099493)

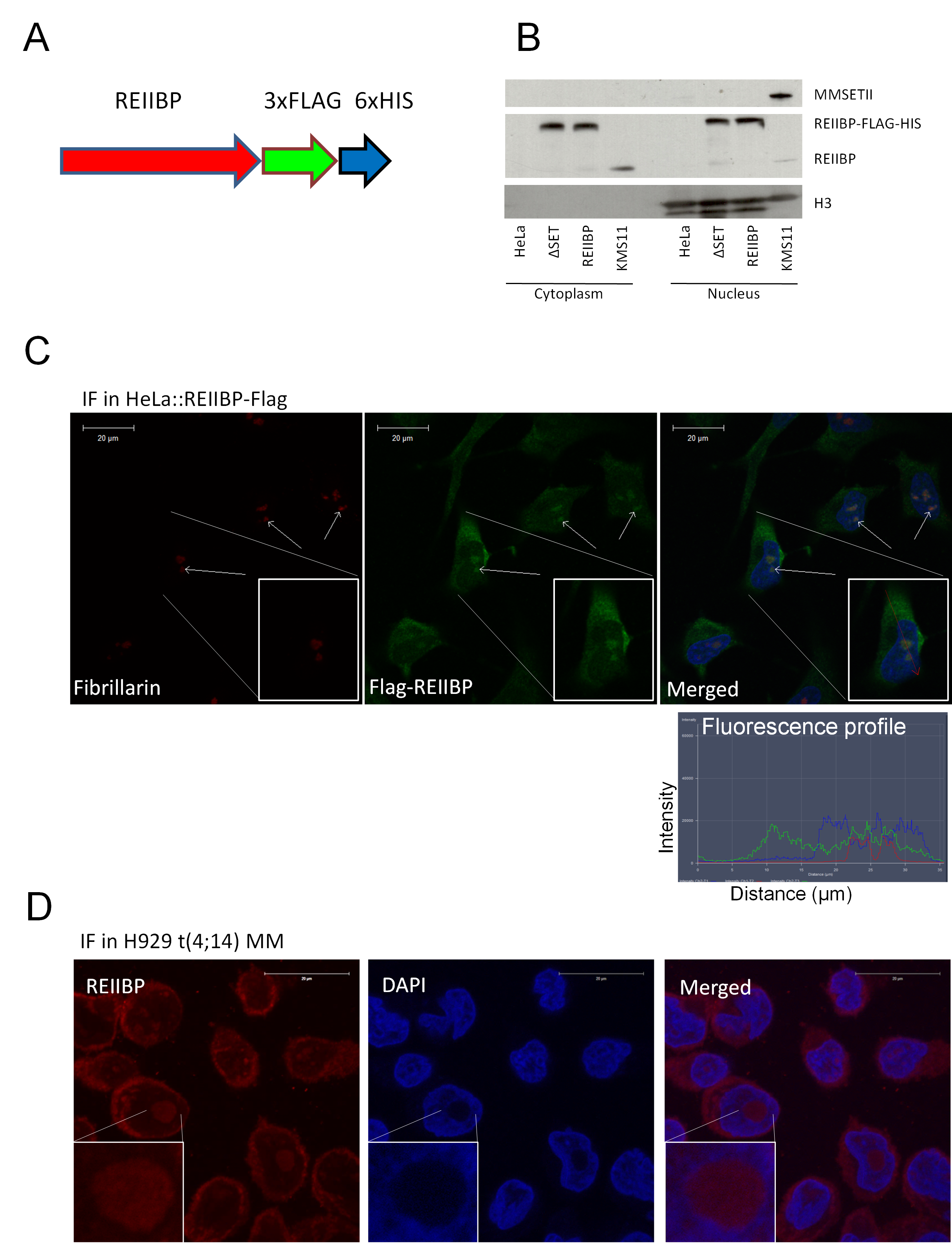

Supplement: Figure S1 — Characterization of HeLa cells transduced with REIIBP constructs. A) Schematic Protein structure of the transduced tagged REIIBP. B) Transduced HeLa lines correctly express REIIBP. The label “REIIBP” represents the HeLa cell line transduced with REIIBP double tagged with 6×HIS-3×FLAG. “ΔSET” is the HeLa cell line transduced with the same construct but with an inactivating mutation in the catalytic SET domain. Cytoplasm and nuclear fractions were isolated and analysed by western blot, using an antibody which recognizes both MMSET II and REIIBP isoforms or an antibody which recognizes Histone 3 (H3). KMS11 is a myeloma cell line used as positive control. The position for each of the respective protein species is indicated. C) IF and confocal analysis. REIIBP-Flag (green) co-localizes with the nucleolus marker Fibrillarin (red) in HeLa cells overexpressing REIIBP-Flag. The merged image includes DAPI (blue) staining. White arrows point to the same cellular region in all three frames. Single cell zoomed in as inset in each frame. Colocalization profile of Fibrillarin and Flag-REIIBP is show under the “merged” frame. The fluorescence profile is generated from the area covered by the red arrow in the merged picture. The blue plot represents the intensity of the DAPI stain; the green plot, the intensity of FLAG and the red the intensity of Fibrillarin. D) IF and confocal analysis on H929 t(4;14) MM cells. REIIBP (red) localizes in the cytoplasm and in the nucleolus. Single nucleolus zoomed in as inset in each frame. (TIF) [file pone.0099493.s001.tif]

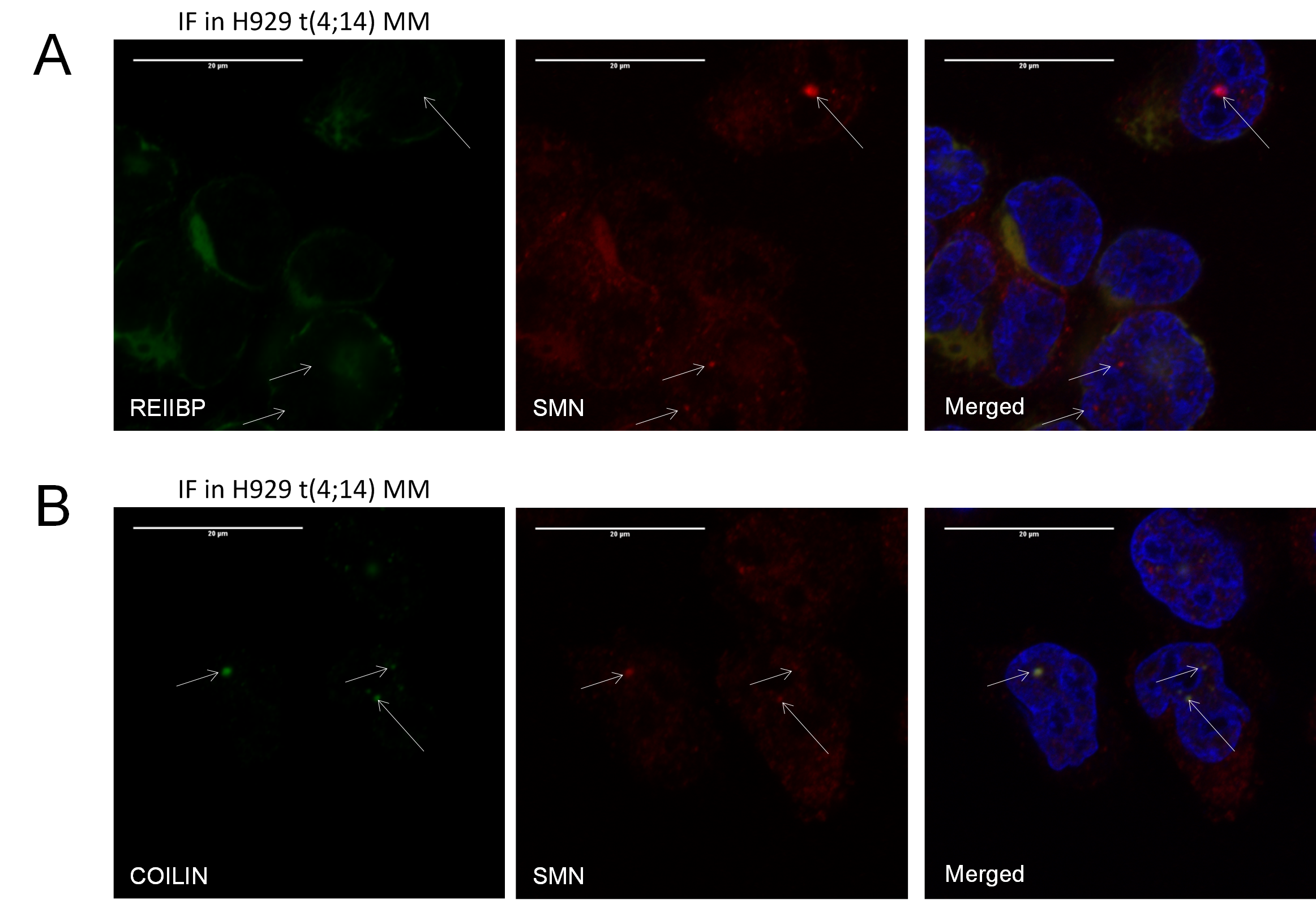

Supplement: Figure S2 — REIIBP co-localizes with the SMN complex in myeloma t(4;14) cells. Immuno fluorescence and confocal analysis on H929 cells. A) REIIBP (green) co-localizes with SMN (red) in the cytoplasm. B) SMN (red) colocalizes with Coilin (green) at level of cajal bodies in the nucleus. The merged images include DAPI (blue) staining and white arrows show the nuclear position for the cajal bodies. Of note the antibody against REIIBP also recognizes the MMSET II isoform. (TIF) [file pone.0099493.s002.tif]

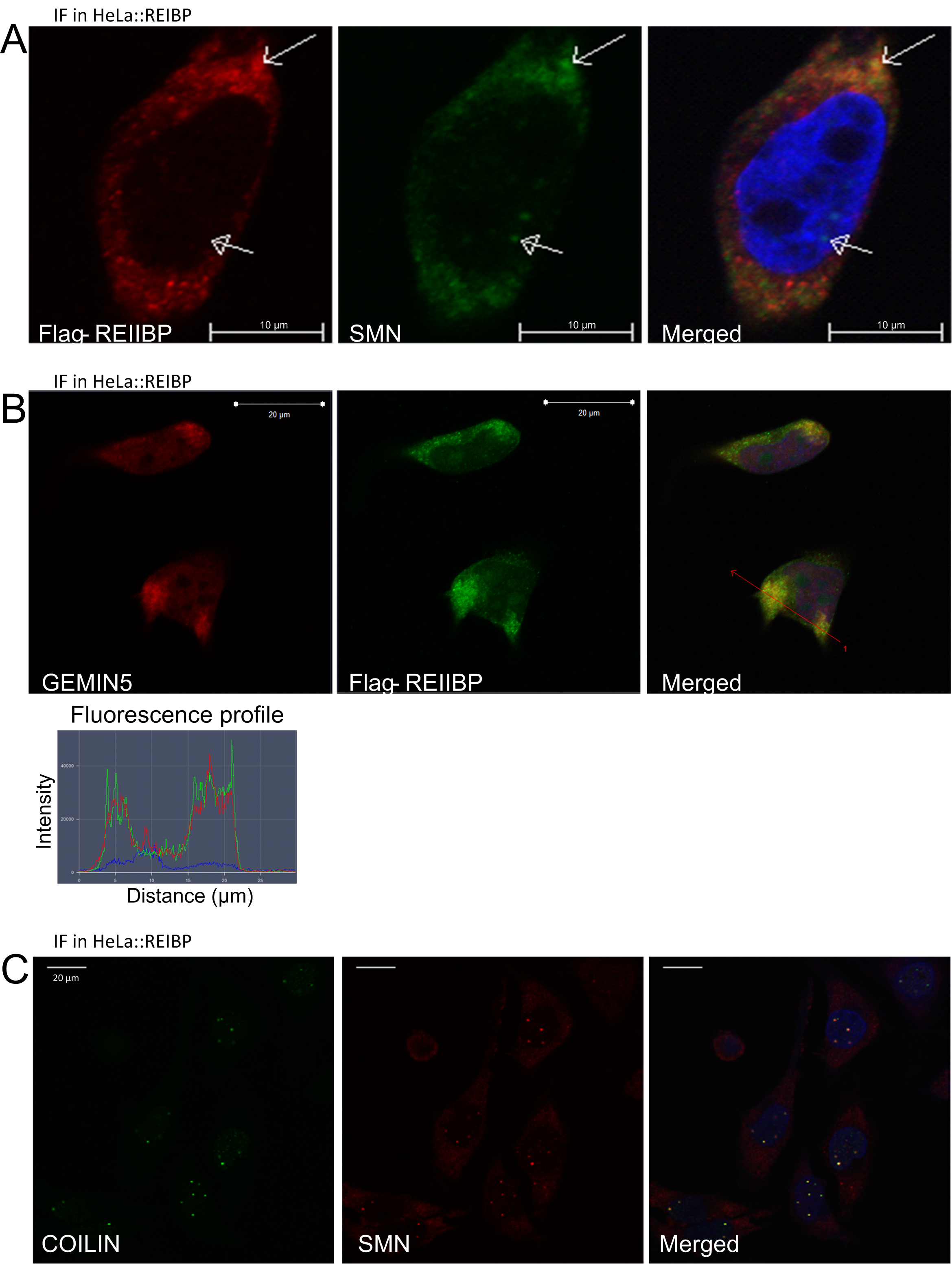

Supplement: Figure S3 — REIIBP-Flag co-localizes with SMN and GEMIN5 in HeLa cells overexpressing REIIBP-Flag. Immuno fluorescence and confocal analysis of HeLa::REIIBP-FLAG cells. A) Colocalization of FLAG (red) with SMN (green). Open arrows show the colocalization signal from the cytoplasm. Closed arrows indicate the nuclear position of Cajal bodies. B) Colocalization of FLAG (green) with GEMIN5 (red). Co-localization profile of GEMIN5 and REIIBP is shown at the bottom. The fluorescence profile is generated from the area covered by the red arrow in the merged picture. The blue plot represents the intensity of the DAPI stain; the green plot, the intensity of FLAG, the red the intensity of GEMIN5. C) SMN (red) co-localizes with Coilin (green) at level of cajal bodies in the nucleus. Merged images in A), B) and C) include DAPI (blue) staining. (TIF) [file pone.0099493.s003.tif]

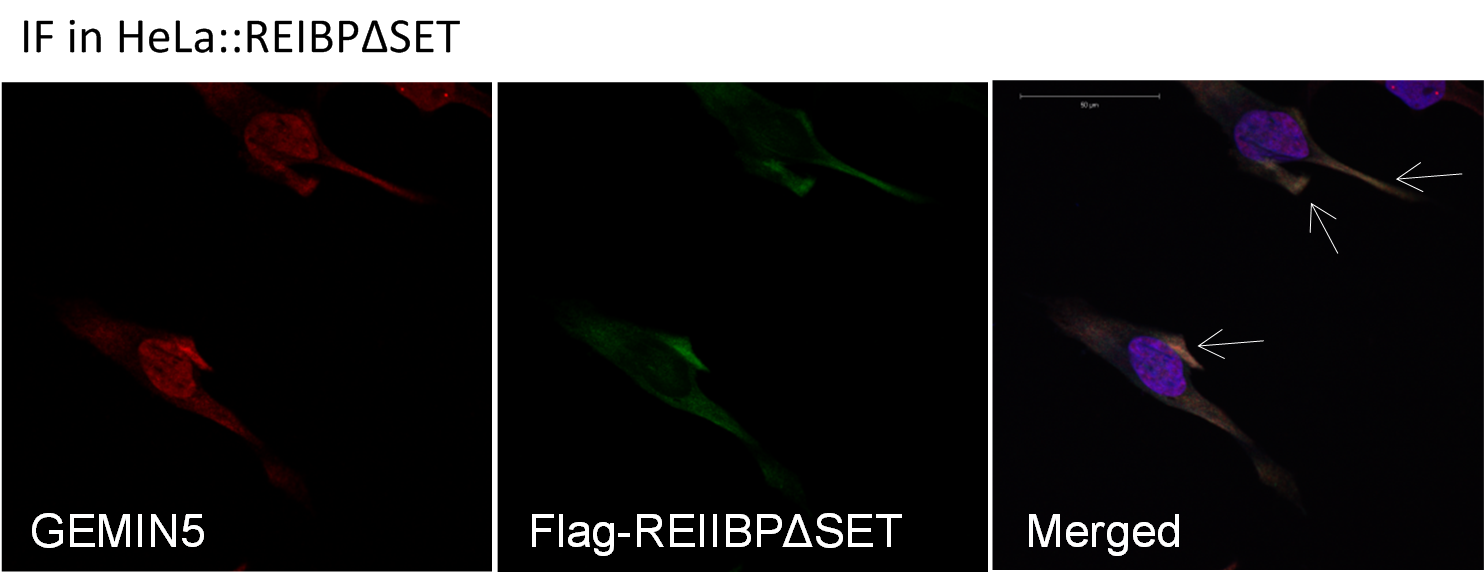

Supplement: Figure S4 — REIIBP-Flag with an inactivating mutation in the SET domain co-localizes with GEMIN5 in HeLa::ΔSET. Immuno fluorescence and confocal analysis of HeLa::ΔSET cells. Colocalization of REIIBPΔSET-FLAG (green) with GEMIN5 (red). Merged image includes DAPI (blue) staining. Arrows show the colocalization signal from the cytoplasm. (TIF) [file pone.0099493.s004.tif]
