# Supplementary material for: A Novel Functional Role for MMSET in RNA Processing Based on the Link Between the REIIBP Isoform and Its Interaction with the SMN Complex
Source: PLoS One. 2014 Jun 12;9(6):e99493. doi: 10.1371/journal.pone.0099493 (PMC4055699; doi:10.1371/journal.pone.0099493)
Supplement: Table S1 — Transcript isoforms alternatively spliced between HeLa and HeLa::REIIBP. RNASeq result showing a list of differently expressed transcripts within genes where the overall expression was unchanged. (DOCX) [file pone.0099493.s005.docx]

**Table S1. Transcript isoforms alternatively spliced between HeLa and HeLa::REIIBP.**

| HeLa | HeLa::REIIBP | log2 fold change | Test stat | p value | q value | TSS Group | Chromosome position |
| --- | --- | --- | --- | --- | --- | --- | --- |
| 0.029902 | 0.556424 | 4.21788 | 4.03695 | 0.00065 | 0.032203 | SLC2A10 | 20:45338125-45364965 |
| 0.108618 | 0.809913 | 2.89851 | 2.46463 | 8.00E-04 | 0.038289 | TNRC6C | 17:75955289-76139049 |
| 0.075885 | 0.519017 | 2.77389 | 4.33167 | 5.00E-05 | 0.003694 | AC097015.1 | 3:53238556-53245177 |
| 0.216032 | 1.09779 | 2.34528 | 3.12188 | 1.00E-04 | 0.006809 | GMDS | 6:1615068-2245926 |
| 1.58824 | 6.45416 | 2.0228 | 2.16556 | 0.00055 | 0.028263 | TJP2 | 9:71735783-71870124 |
| 0.366997 | 1.46231 | 1.9944 | 2.75963 | 1.00E-04 | 0.006809 | AP006621.5 | 11:777577-784516 |
| 1.31419 | 5.03108 | 1.9367 | 2.91459 | 0.00035 | 0.019282 | MRPL42 | 12:93861259-93906707 |
| 0.251863 | 0.895892 | 1.83069 | 2.23512 | 5.00E-04 | 0.026095 | FAM125B | 9:129089127-129269320 |
| 1.09307 | 3.65224 | 1.7404 | 2.95728 | 5.00E-05 | 0.003694 | SC5DL | 11:121163161-121184104 |
| 0.288086 | 0.894957 | 1.63532 | 2.48513 | 8.00E-04 | 0.038289 | HCG15 | 6:28953590-28958762 |
| 0.252897 | 0.778478 | 1.6221 | 2.25773 | 0.00075 | 0.036334 | EHHADH | 3:184880658-185000701 |
| 0.583824 | 1.79173 | 1.61775 | 2.57905 | 2.00E-04 | 0.01224 | RIMKLBP2 | 1:219259943-219389424 |
| 0.276373 | 0.816091 | 1.56211 | 3.0086 | 5.00E-05 | 0.003694 | RP11-33B1.2 | 4:120352184-120375785 |
| 0.788805 | 2.23708 | 1.50387 | 2.26559 | 0.0011 | 0.049266 | CENPH | 5:68481346-68511652 |
| 0.399918 | 1.12268 | 1.48918 | 2.64597 | 0.00025 | 0.014623 | ZNF286B | 17:18538318-18586241 |
| 0.889256 | 2.45211 | 1.46335 | 2.61536 | 0.00035 | 0.019282 | UBN2 | 7:138915101-138993304 |
| 0.797576 | 2.17438 | 1.44691 | 2.17832 | 2.00E-04 | 0.01224 | AL589743.1 | 14:19650017-19925348 |
| 0.844802 | 2.30255 | 1.44655 | 2.53505 | 0.00035 | 0.019282 | MXD1 | 2:70120691-70170079 |
| 0.79033 | 2.12693 | 1.42825 | 2.12316 | 7.00E-04 | 0.034173 | STRIP1 | 1:110574198-110617263 |
| 0.455989 | 1.21921 | 1.41887 | 2.3525 | 0.00025 | 0.014623 | PPM1L | 3:160473377-160796888 |
| 1.7346 | 4.43681 | 1.35492 | 2.76832 | 5.00E-05 | 0.003694 | ENTPD1 | 10:97454773-97849995 |
| 0.372235 | 0.937324 | 1.33234 | 2.08984 | 7.00E-04 | 0.034173 | ZNF184 | 6:27418366-27448120 |
| 0.766372 | 1.92073 | 1.32554 | 2.45579 | 3.00E-04 | 0.016984 | HELB | 12:66696282-67197966 |
| 0.903608 | 2.20211 | 1.28512 | 3.46051 | 5.00E-05 | 0.003694 | PRTG | 15:55903743-56035288 |
| 0.562537 | 1.32387 | 1.23474 | 2.64183 | 5.00E-05 | 0.003694 | AC093724.2 | 2:132438891-132524973 |
| 0.63973 | 1.49773 | 1.22724 | 2.15179 | 0.00095 | 0.04404 | RFC3 | 13:34392185-34540695 |
| 2.36407 | 5.4863 | 1.21456 | 2.0986 | 0.0011 | 0.049266 | NOC3L | 10:95753745-96122732 |
| 0.263558 | 0.603349 | 1.19487 | 2.66949 | 5.00E-05 | 0.003694 | SNORD75 | 2:42653413-42668864 |
| 0.358586 | 0.818853 | 1.19128 | 2.71031 | 5.00E-05 | 0.003694 | RAB3B | 1:52373627-52456436 |
| 2.8835 | 6.34015 | 1.13669 | 2.50607 | 5.00E-05 | 0.003694 | EXOC6 | 10:94590934-94819368 |
| 1.13499 | 2.48731 | 1.13191 | 2.78898 | 5.00E-05 | 0.003694 | AC004383.4 | X:133677193-133683669 |
| 1.20247 | 2.58763 | 1.10563 | 3.36694 | 5.00E-05 | 0.003694 | RP1-167A14.2 | 6:167383113-167412609 |
| 7.33391 | 15.1459 | 1.04628 | 2.13013 | 6.00E-04 | 0.030221 | PHF6 | X:133507165-133562820 |
| 0.388854 | 0.799372 | 1.03964 | 2.08438 | 3.00E-04 | 0.016984 | AC009404.2 | 2:118590278-118599234 |
| 1.13602 | 2.28031 | 1.00523 | 2.8651 | 5.00E-05 | 0.003694 | Metazoa_SRP | 6:20400241-20495487 |
| 11.449 | 22.8182 | 0.994965 | 2.33042 | 0.00035 | 0.019282 | BMI1 | 10:22604902-22620413 |
| 1.60134 | 3.17573 | 0.987809 | 2.00541 | 0.00055 | 0.028263 | ACVR1B | 12:52345450-52391254 |
| 0.414814 | 0.818537 | 0.980584 | 1.82139 | 0.0011 | 0.049266 | AC139530.1 | 17:79642930-79647625 |
| 2.49709 | 4.90382 | 0.973661 | 2.07467 | 0.00065 | 0.032203 | CHST10 | 2:101008214-101034118 |
| 5.65752 | 10.9581 | 0.953755 | 2.81912 | 5.00E-05 | 0.003694 | ETNK1 | 12:22778008-22843808 |
| 1.57194 | 3.0218 | 0.942861 | 2.05883 | 0.00055 | 0.028263 | PLEKHA8 | 7:30067019-30170096 |
| 1.48077 | 2.72118 | 0.877885 | 1.95703 | 0.001 | 0.045723 | USP30 | 12:109460893-109525988 |
| 1.92957 | 3.53942 | 0.875232 | 2.07571 | 9.00E-04 | 0.042122 | TXNDC16 | 14:52897136-53019266 |
| 7.56081 | 13.8675 | 0.875092 | 1.81185 | 9.00E-04 | 0.042122 | KRI1 | 19:10654570-10676713 |
| 5.53457 | 10.0177 | 0.856005 | 2.08189 | 0.00045 | 0.023859 | MTF2 | 1:93544767-93609231 |
| 2.03989 | 3.5425 | 0.79628 | 2.06557 | 0.00085 | 0.040287 | ENPP1 | 6:132129155-132216296 |
| 1.45814 | 2.50773 | 0.782251 | 2.01658 | 0.00075 | 0.036334 | INSR | 19:7112265-7294045 |
| 0.705278 | 1.19262 | 0.757876 | 2.04494 | 0.00065 | 0.032203 | RP4-535B20.1 | 1:65518715-65533884 |
| 15.6116 | 26.3928 | 0.75753 | 2.92576 | 5.00E-05 | 0.003694 | PANK3 | 5:167975291-168006605 |
| 4.84194 | 7.96142 | 0.717441 | 2.03253 | 6.00E-04 | 0.030221 | TMX4 | 20:7957873-8000476 |
| 10.4794 | 16.7543 | 0.676986 | 1.89632 | 4.00E-04 | 0.021477 | DDX10 | 11:108535751-108811727 |
| 5.37593 | 8.45835 | 0.653863 | 2.1004 | 4.00E-04 | 0.021477 | SPTLC2 | 14:77972338-78083116 |
| 12.7996 | 20.0755 | 0.64933 | 1.85301 | 8.00E-04 | 0.038289 | RRP1 | 21:45209393-45225174 |
| 4.54168 | 6.96772 | 0.617458 | 2.02539 | 0.0011 | 0.049266 | UBXN7 | 3:196074531-196160242 |
| 29.5207 | 20.516 | -0.52498 | -1.80636 | 0.001 | 0.045723 | POFUT1 | 20:30795682-30826470 |
| 128.249 | 87.0551 | -0.55894 | -1.98759 | 7.00E-04 | 0.034173 | TFRC | 3:195754053-195809060 |
| 32.9848 | 22.1784 | -0.57265 | -1.88021 | 0.00095 | 0.04404 | KIF1C | 17:4901242-4931696 |
| 83.7427 | 55.5742 | -0.59155 | -2.14122 | 4.00E-04 | 0.021477 | STT3B | 3:31574129-31679116 |
| 66.3892 | 42.8621 | -0.63125 | -2.12699 | 0.00105 | 0.047429 | CAPZA2 | 7:116451123-116559315 |
| 19.7067 | 12.6025 | -0.64498 | -2.0407 | 8.00E-04 | 0.038289 | ARRDC3 | 5:90664540-90680633 |
| 24.9958 | 15.93 | -0.64994 | -2.09775 | 0.00035 | 0.019282 | ADAM10 | 15:58887402-59042177 |
| 41.833 | 26.5558 | -0.65561 | -2.12478 | 5.00E-05 | 0.003694 | BUB1B | 15:40453223-40569688 |
| 36.3901 | 22.8125 | -0.67373 | -2.33051 | 1.00E-04 | 0.006809 | IL6ST | 5:55230922-55290821 |
| 10.5948 | 6.63851 | -0.67442 | -2.04439 | 0.00045 | 0.023859 | HERC2 | 15:28356036-28567298 |
| 38.6037 | 24.0542 | -0.68245 | -2.02744 | 0.00045 | 0.023859 | LAMC1 | 1:182992596-183114844 |
| 7.14603 | 4.4262 | -0.69107 | -2.12432 | 0.00015 | 0.009587 | SH3PXD2B | 5:171752184-171881527 |
| 21.0705 | 12.9076 | -0.707 | -2.38199 | 5.00E-05 | 0.003694 | SHCBP1 | 16:46614465-46655538 |
| 1.92483 | 1.1786 | -0.70766 | -2.2067 | 5.00E-04 | 0.026095 | RP13-143G15.3 | 6:136172833-136546733 |
| 8.58035 | 5.18987 | -0.72534 | -1.94999 | 0.00065 | 0.032203 | CUX1 | 7:101459218-101927309 |
| 23.2228 | 13.9486 | -0.73543 | -2.45226 | 1.00E-04 | 0.006809 | RHOBTB3 | 5:95036952-95160087 |
| 7.63203 | 4.45237 | -0.77749 | -2.23226 | 0.00025 | 0.014623 | HGSNAT | 8:42995555-43057998 |
| 435.966 | 252.72 | -0.78668 | -2.4356 | 5.00E-05 | 0.003694 | MALAT1 | 11:65265164-65276596 |
| 9.59688 | 5.53412 | -0.79421 | -2.47333 | 5.00E-05 | 0.003694 | UBE2L2 | 12:20514665-20909803 |
| 4.96312 | 2.81669 | -0.81725 | -2.08149 | 9.00E-04 | 0.042122 | LPCAT2 | 16:55542796-55620582 |
| 8.72166 | 4.93352 | -0.82199 | -2.28886 | 5.00E-05 | 0.003694 | TANC2 | 17:61012762-61509572 |
| 16.2925 | 9.19772 | -0.82486 | -2.14714 | 0.00025 | 0.014623 | C16orf58 | 16:31494322-31520630 |
| 218.616 | 123.058 | -0.82906 | -2.40998 | 5.00E-05 | 0.003694 | TNFRSF12A | 16:3056166-3072384 |
| 31.185 | 17.4337 | -0.83897 | -2.08917 | 0.001 | 0.045723 | LRFN4 | 11:66615038-66725885 |
| 1.69585 | 0.934994 | -0.85898 | -2.63505 | 5.00E-05 | 0.003694 | AC003989.4 | 7:110302744-111202586 |
| 0.937155 | 0.503929 | -0.89507 | -2.49762 | 5.00E-05 | 0.003694 | RP11-608O21.1 | 4:19173957-19911315 |
| 5.77764 | 3.08368 | -0.90583 | -2.29093 | 2.00E-04 | 0.01224 | MIR31HG | 9:21439190-21592123 |
| 8.73238 | 4.63367 | -0.91422 | -2.14598 | 5.00E-05 | 0.003694 | RNF213 | 17:78234660-78411886 |
| 3.96517 | 2.05471 | -0.94845 | -2.09788 | 3.00E-04 | 0.016984 | MAPKBP1 | 15:42066631-42120134 |
| 6.07908 | 3.14076 | -0.95274 | -1.98598 | 0.00035 | 0.019282 | LINC00473 | 6:165740622-166401690 |
| 27.7577 | 14.2339 | -0.96355 | -3.0727 | 5.00E-05 | 0.003694 | ITPR1 | 3:4535031-4928977 |
| 1.60999 | 0.823584 | -0.96706 | -3.25865 | 5.00E-05 | 0.003694 | AC012501.2 | 2:154264880-154449801 |
| 1.03887 | 0.526783 | -0.97973 | -2.43407 | 2.00E-04 | 0.01224 | IL15 | 4:142556729-142658723 |
| 4.13527 | 2.09609 | -0.98028 | -1.99382 | 8.00E-04 | 0.038289 | PAPPA | 9:118915724-119164968 |
| 12.6538 | 6.37557 | -0.98894 | -2.13638 | 1.00E-04 | 0.006809 | ZFP106 | 15:42704594-42783321 |
| 1.85666 | 0.930685 | -0.99635 | -2.83073 | 5.00E-05 | 0.003694 | RP11-25K19.1 | 8:59717318-60034034 |
| 2.46287 | 1.22725 | -1.00491 | -2.12938 | 0.00055 | 0.028263 | PLXND1 | 3:129273929-129325661 |
| 5.59751 | 2.74372 | -1.02865 | -2.96154 | 5.00E-05 | 0.003694 | T | 6:166568398-166614229 |
| 7.40793 | 3.6298 | -1.02918 | -1.98909 | 0.00055 | 0.028263 | SERPINI1 | 3:167453030-167543356 |
| 1.18217 | 0.569805 | -1.05289 | -2.7532 | 5.00E-05 | 0.003694 | SNORD74 | 21:17442841-17986756 |
| 8.24495 | 3.95512 | -1.05979 | -2.10515 | 0.00065 | 0.032203 | U1 | 1:144534037-144534914 |
| 1.43636 | 0.678662 | -1.08165 | -2.694 | 5.00E-05 | 0.003694 | GRM7-AS3 | 3:6532157-7783215 |
| 42.4803 | 20.0061 | -1.08635 | -1.94209 | 7.00E-04 | 0.034173 | C7orf50 | 7:1036609-1177896 |
| 0.940963 | 0.426215 | -1.14256 | -3.05453 | 5.00E-05 | 0.003694 | U6 | X:115522174-115545654 |
| 0.733828 | 0.329919 | -1.15333 | -2.11405 | 0.001 | 0.045723 | SLFN5 | 17:33570054-33600674 |
| 39.2903 | 17.6452 | -1.1549 | -2.12697 | 6.00E-04 | 0.030221 | TUBB3 | 16:89884586-90005169 |
| 3.03352 | 1.36038 | -1.15698 | -2.13143 | 0.00095 | 0.04404 | IFNA1 | 9:21439190-21592123 |
| 2.42875 | 1.08628 | -1.16082 | -2.69478 | 5.00E-05 | 0.003694 | TMED11P | 4:1050037-1202750 |
| 87.8869 | 38.5191 | -1.19007 | -2.15093 | 5.00E-05 | 0.003694 | AHNAK | 11:62200934-62360492 |
| 2.49664 | 1.07309 | -1.21822 | -2.20409 | 1.00E-04 | 0.006809 | TRPM2 | 21:45770045-45862964 |
| 3.31933 | 1.40354 | -1.24183 | -3.67682 | 5.00E-05 | 0.003694 | RP11-65J21.3 | 16:14396104-14428707 |
| 0.872396 | 0.366148 | -1.25256 | -2.20096 | 0.00105 | 0.047429 | RP11-807H7.2 | 4:66565996-66573971 |
| 0.63893 | 0.259828 | -1.2981 | -2.34422 | 0.00025 | 0.014623 | AL136985.1 | 1:59226696-59232071 |
| 17.6707 | 7.04729 | -1.32622 | -2.55935 | 5.00E-05 | 0.003694 | MYOCD | 17:12567328-12921504 |
| 1.3304 | 0.526355 | -1.33775 | -3.70237 | 5.00E-05 | 0.003694 | RP11-93K7.1 | 6:101841541-102518607 |
| 1.36713 | 0.538604 | -1.34386 | -2.1791 | 0.00035 | 0.019282 | SPON2 | 4:1050037-1202750 |
| 0.844219 | 0.33162 | -1.34808 | -2.34728 | 2.00E-04 | 0.01224 | RP11-810P12.1 | 11:61776661-61782055 |
| 0.733589 | 0.288013 | -1.34884 | -3.17121 | 5.00E-05 | 0.003694 | AC108865.1 | 4:187769246-187784539 |
| 1.03545 | 0.402173 | -1.36437 | -2.73164 | 5.00E-05 | 0.003694 | AL139147.1 | 1:66999065-67213982 |
| 0.800952 | 0.307198 | -1.38255 | -2.57023 | 1.00E-04 | 0.006809 | NA | 21:16741127-16816748 |
| 5.8869 | 2.17886 | -1.43393 | -2.495 | 5.00E-05 | 0.003694 | SMTN | 22:31460090-31500743 |
| 1.32913 | 0.485291 | -1.45356 | -2.552 | 1.00E-04 | 0.006809 | ZNF365 | 10:64133950-64431771 |
| 1.27689 | 0.448962 | -1.50796 | -5.25397 | 5.00E-05 | 0.003694 | AC087433.1 | 15:47177562-47252145 |
| 0.63066 | 0.221234 | -1.51129 | -3.07399 | 5.00E-05 | 0.003694 | U8 | 2:186526146-186534747 |
| 18.0846 | 6.29293 | -1.52296 | -2.66445 | 5.00E-05 | 0.003694 | L1CAM | X:153126967-153200676 |
| 4.0235 | 1.3104 | -1.61844 | -2.38436 | 2.00E-04 | 0.01224 | SLC44A2 | 19:10713132-10755235 |
| 1.89823 | 0.606862 | -1.64522 | -2.63615 | 1.00E-04 | 0.006809 | ZNF808 | 19:52956828-53090427 |
| 1.81713 | 0.561997 | -1.69302 | -2.56429 | 2.00E-04 | 0.01224 | CTD-2139B15.5 | 5:17367400-17375786 |
| 7.25806 | 2.06583 | -1.81287 | -3.52855 | 5.00E-05 | 0.003694 | RPS6KA2 | 6:166822670-167370679 |
| 6.82302 | 1.80508 | -1.91835 | -2.02698 | 0.00095 | 0.04404 | NF2 | 22:29999544-30094587 |
| 0.775329 | 0.19375 | -2.00061 | -2.3632 | 5.00E-05 | 0.003694 | HMCN1 | 1:185703565-186160170 |
| 5.8199 | 1.43676 | -2.01817 | -2.17194 | 7.00E-04 | 0.034173 | ARHGEF1 | 19:42387227-42434302 |
| 0.526637 | 0.125309 | -2.07132 | -2.66274 | 0.00105 | 0.047429 | C8orf42 | 8:439802-495781 |

RNASeq result showing a list of differently expressed transcripts within genes where the overall expression was unchanged.
