# Supplementary material for: A Novel Functional Role for MMSET in RNA Processing Based on the Link Between the REIIBP Isoform and Its Interaction with the SMN Complex
Source: PLoS One. 2014 Jun 12;9(6):e99493. doi: 10.1371/journal.pone.0099493 (PMC4055699; doi:10.1371/journal.pone.0099493)
Supplement: Table S2 — Gene ontology analysis of genes overexpressed in HeLa::REIIBP. List copiled using DAVID bionformatic resources (http://david.abcc.ncifcrf.gov/). Fold enrichment measures the magnitude of enrichment compared to the human genome. Fold enrichment >1.5 was considered as interesting. The percentage is the total number of genes involved in given term divided by the total number of input gene. P-value examine the significance of gene-term enrichment. P value <0.05 was considered significant. (DOCX) [file pone.0099493.s006.docx]

**Table S2. Gene ontology analysis of genes overexpressed in HeLa::REIIBP.**

| [Term](http://david.abcc.ncifcrf.gov/chartReport.jsp?visited=yes&d-16544-s=2&cbBenjamini=true&rowids=&count=2&d-16544-o=2&cbFC=true&d-16544-p=1&annot=26&ease=0.1&numRecords=1000&heading=) | [Count](http://david.abcc.ncifcrf.gov/chartReport.jsp?visited=yes&d-16544-s=5&cbBenjamini=true&rowids=&count=2&d-16544-o=1&cbFC=true&d-16544-p=1&annot=26&ease=0.1&numRecords=1000&heading=) | [%](http://david.abcc.ncifcrf.gov/chartReport.jsp?visited=yes&d-16544-s=6&cbBenjamini=true&rowids=&count=2&d-16544-o=1&cbFC=true&d-16544-p=1&annot=26&ease=0.1&numRecords=1000&heading=) | [p-Value](http://david.abcc.ncifcrf.gov/chartReport.jsp?visited=yes&d-16544-s=7&cbBenjamini=true&rowids=&count=2&d-16544-o=1&cbFC=true&d-16544-p=1&annot=26&ease=0.1&numRecords=1000&heading=) | [Fold Enrichment](http://david.abcc.ncifcrf.gov/chartReport.jsp?visited=yes&d-16544-s=8&cbBenjamini=true&rowids=&count=2&d-16544-o=1&cbFC=true&d-16544-p=1&annot=26&ease=0.1&numRecords=1000&heading=) |
| --- | --- | --- | --- | --- |
| [nervous system development](http://www.ebi.ac.uk/QuickGO/GTerm?id=GO:0007399) | 134 | 12.3 | 6.8E-19 | 2.2 |
| [system development](http://www.ebi.ac.uk/QuickGO/GTerm?id=GO:0048731) | 225 | 20.7 | 1.8E-18 | 1.7 |
| [anatomical structure development](http://www.ebi.ac.uk/QuickGO/GTerm?id=GO:0048856) | 238 | 21.9 | 2.6E-18 | 1.7 |
| [multicellular organismal development](http://www.ebi.ac.uk/QuickGO/GTerm?id=GO:0007275) | 260 | 23.9 | 4.3E-18 | 1.6 |
| [developmental process](http://www.ebi.ac.uk/QuickGO/GTerm?id=GO:0032502) | 275 | 25.3 | 5.0E-17 | 1.6 |
| [embryonic development](http://www.ebi.ac.uk/QuickGO/GTerm?id=GO:0009790) | 83 | 7.6 | 1.0E-15 | 2.6 |
| [regionalization](http://www.ebi.ac.uk/QuickGO/GTerm?id=GO:0003002) | 45 | 4.1 | 1.2E-15 | 4.1 |
| [anatomical structure morphogenesis](http://www.ebi.ac.uk/QuickGO/GTerm?id=GO:0009653) | 135 | 12.4 | 1.3E-15 | 2.0 |
| [embryonic morphogenesis](http://www.ebi.ac.uk/QuickGO/GTerm?id=GO:0048598) | 57 | 5.2 | 2.1E-15 | 3.3 |
| [pattern specification process](http://www.ebi.ac.uk/QuickGO/GTerm?id=GO:0007389) | 52 | 4.8 | 5.7E-15 | 3.5 |
| [generation of neurons](http://www.ebi.ac.uk/QuickGO/GTerm?id=GO:0048699) | 80 | 7.4 | 1.1E-14 | 2.6 |
| [neurogenesis](http://www.ebi.ac.uk/QuickGO/GTerm?id=GO:0022008) | 83 | 7.6 | 2.5E-14 | 2.5 |
| [anterior/posterior pattern formation](http://www.ebi.ac.uk/QuickGO/GTerm?id=GO:0009952) | 35 | 3.2 | 1.7E-13 | 4.5 |
| [embryonic organ morphogenesis](http://www.ebi.ac.uk/QuickGO/GTerm?id=GO:0048562) | 34 | 3.1 | 2.0E-13 | 4.6 |
| [chordate embryonic development](http://www.ebi.ac.uk/QuickGO/GTerm?id=GO:0043009) | 56 | 5.2 | 2.2E-13 | 3.0 |
| [cellular developmental process](http://www.ebi.ac.uk/QuickGO/GTerm?id=GO:0048869) | 166 | 15.3 | 2.4E-13 | 1.7 |
| [embryonic development ending in birth or egg hatching](http://www.ebi.ac.uk/QuickGO/GTerm?id=GO:0009792) | 56 | 5.2 | 3.2E-13 | 3.0 |
| [cell differentiation](http://www.ebi.ac.uk/QuickGO/GTerm?id=GO:0030154) | 160 | 14.7 | 5.3E-13 | 1.8 |
| [embryonic organ development](http://www.ebi.ac.uk/QuickGO/GTerm?id=GO:0048568) | 38 | 3.5 | 7.8E-13 | 4.0 |
| [regulation of gene expression](http://www.ebi.ac.uk/QuickGO/GTerm?id=GO:0010468) | 241 | 22.2 | 1.4E-12 | 1.5 |
| [embryonic skeletal system development](http://www.ebi.ac.uk/QuickGO/GTerm?id=GO:0048706) | 25 | 2.3 | 1.9E-12 | 5.8 |
| [regulation of transcription](http://www.ebi.ac.uk/QuickGO/GTerm?id=GO:0045449) | 223 | 20.5 | 3.2E-12 | 1.5 |
| [organ morphogenesis](http://www.ebi.ac.uk/QuickGO/GTerm?id=GO:0009887) | 75 | 6.9 | 3.7E-12 | 2.4 |
| [neuron differentiation](http://www.ebi.ac.uk/QuickGO/GTerm?id=GO:0030182) | 63 | 5.8 | 9.1E-12 | 2.6 |
| [embryonic skeletal system morphogenesis](http://www.ebi.ac.uk/QuickGO/GTerm?id=GO:0048704) | 21 | 1.9 | 1.1E-11 | 6.6 |
| [regulation of nitrogen compound metabolic process](http://www.ebi.ac.uk/QuickGO/GTerm?id=GO:0051171) | 234 | 21.5 | 4.2E-11 | 1.5 |
| [regulation of transcription, DNA-dependent](http://www.ebi.ac.uk/QuickGO/GTerm?id=GO:0006355) | 163 | 15.0 | 4.5E-11 | 1.6 |
| [regulation of nucleobase, nucleoside, nucleotide and nucleic acid metabolic process](http://www.ebi.ac.uk/QuickGO/GTerm?id=GO:0019219) | 232 | 21.3 | 5.2E-11 | 1.5 |
| [regulation of RNA metabolic process](http://www.ebi.ac.uk/QuickGO/GTerm?id=GO:0051252) | 165 | 15.2 | 7.5E-11 | 1.6 |
| [regulation of macromolecule biosynthetic process](http://www.ebi.ac.uk/QuickGO/GTerm?id=GO:0010556) | 232 | 21.3 | 9.3E-11 | 1.5 |
| [transcription](http://www.ebi.ac.uk/QuickGO/GTerm?id=GO:0006350) | 183 | 16.8 | 1.9E-10 | 1.6 |
| [regulation of cellular biosynthetic process](http://www.ebi.ac.uk/QuickGO/GTerm?id=GO:0031326) | 238 | 21.9 | 2.0E-10 | 1.4 |
| [organ development](http://www.ebi.ac.uk/QuickGO/GTerm?id=GO:0048513) | 158 | 14.5 | 2.3E-10 | 1.6 |
| [skeletal system morphogenesis](http://www.ebi.ac.uk/QuickGO/GTerm?id=GO:0048705) | 27 | 2.5 | 3.5E-10 | 4.3 |
| [multicellular organismal process](http://www.ebi.ac.uk/QuickGO/GTerm?id=GO:0032501) | 319 | 29.3 | 3.8E-10 | 1.3 |
| [regulation of biosynthetic process](http://www.ebi.ac.uk/QuickGO/GTerm?id=GO:0009889) | 238 | 21.9 | 3.9E-10 | 1.4 |
| [regulation of metabolic process](http://www.ebi.ac.uk/QuickGO/GTerm?id=GO:0019222) | 278 | 25.6 | 6.2E-10 | 1.4 |
| [regulation of cellular metabolic process](http://www.ebi.ac.uk/QuickGO/GTerm?id=GO:0031323) | 268 | 24.7 | 7.3E-10 | 1.4 |
| [regulation of macromolecule metabolic process](http://www.ebi.ac.uk/QuickGO/GTerm?id=GO:0060255) | 253 | 23.3 | 2.2E-9 | 1.4 |
| [tube development](http://www.ebi.ac.uk/QuickGO/GTerm?id=GO:0035295) | 37 | 3.4 | 5.1E-9 | 3.0 |
| [regulation of primary metabolic process](http://www.ebi.ac.uk/QuickGO/GTerm?id=GO:0080090) | 252 | 23.2 | 9.4E-9 | 1.4 |
| [skeletal system development](http://www.ebi.ac.uk/QuickGO/GTerm?id=GO:0001501) | 45 | 4.1 | 2.3E-8 | 2.5 |
| [biological regulation](http://www.ebi.ac.uk/QuickGO/GTerm?id=GO:0065007) | 492 | 45.3 | 3.0E-8 | 1.2 |
| [cell development](http://www.ebi.ac.uk/QuickGO/GTerm?id=GO:0048468) | 71 | 6.5 | 5.0E-8 | 2.0 |
| [neuron development](http://www.ebi.ac.uk/QuickGO/GTerm?id=GO:0048666) | 46 | 4.2 | 5.1E-8 | 2.4 |

List copiled using DAVID bionformatic resources (<http://david.abcc.ncifcrf.gov/>). Fold enrichment measures the magnitude of enrichment compared to the human genome. Fold enrichment > 1.5 was considered as interesting. The percentage is the total number of genes involved in given term divided by the total number of input gene. P-value examine the significance of gene-term enrichment. P value <0.05 was considered significant.
