# Supplementary material for: A Novel Functional Role for MMSET in RNA Processing Based on the Link Between the REIIBP Isoform and Its Interaction with the SMN Complex
Source: PLoS One. 2014 Jun 12;9(6):e99493. doi: 10.1371/journal.pone.0099493 (PMC4055699; doi:10.1371/journal.pone.0099493)
Supplement: Table S3 — Gene pathway analysis of genes overexpressed in HeLa::REIIBP cells. KEGG_PATHWAY analysis of genes overexpressed in HeLa::REIIBP cells. The list was copiled using DAVID bionformatic resources (http://david.abcc.ncifcrf.gov/). Fold enrichment measures the magnitude of enrichment compare to human genome. Fold enrichment >1.5 was considered as interesting. The percentage is the total number of genes involved in a given term divided by the total number of input genes. P-values examine the significance of gene-term enrichment. P value <0.05 was considered significant. PANTHER and REACTOME pathway analysis gave similar results. (DOCX) [file pone.0099493.s007.docx]

**Table S3. Gene pathway analysis of genes overexpressed in HeLa::REIIBP cells.**

| [Term](http://david.abcc.ncifcrf.gov/chartReport.jsp?visited=yes&d-16544-s=2&cbBenjamini=true&rowids=&count=2&d-16544-o=2&cbFC=true&d-16544-p=1&annot=47&ease=0.1&numRecords=1000&heading=) | [Count](http://david.abcc.ncifcrf.gov/chartReport.jsp?visited=yes&d-16544-s=5&cbBenjamini=true&rowids=&count=2&d-16544-o=1&cbFC=true&d-16544-p=1&annot=47&ease=0.1&numRecords=1000&heading=) | [%](http://david.abcc.ncifcrf.gov/chartReport.jsp?visited=yes&d-16544-s=6&cbBenjamini=true&rowids=&count=2&d-16544-o=1&cbFC=true&d-16544-p=1&annot=47&ease=0.1&numRecords=1000&heading=) | [p-Value](http://david.abcc.ncifcrf.gov/chartReport.jsp?visited=yes&d-16544-s=7&cbBenjamini=true&rowids=&count=2&d-16544-o=1&cbFC=true&d-16544-p=1&annot=47&ease=0.1&numRecords=1000&heading=) | [Fold Enrichment](http://david.abcc.ncifcrf.gov/chartReport.jsp?visited=yes&d-16544-s=8&cbBenjamini=true&rowids=&count=2&d-16544-o=1&cbFC=true&d-16544-p=1&annot=47&ease=0.1&numRecords=1000&heading=) |
| --- | --- | --- | --- | --- |
| [Axon guidance](http://david.abcc.ncifcrf.gov/kegg.jsp?path=hsa04360$Axon%20guidance&termId=470038851&source=kegg) | 21 | 1.9 | 1.2E-5 | 3.0 |
| [Systemic lupus erythematosus](http://david.abcc.ncifcrf.gov/kegg.jsp?path=hsa05322$Systemic%20lupus%20erythematosus&termId=470038916&source=kegg) | 13 | 1.2 | 6.0E-3 | 2.5 |
| [Cell adhesion molecules (CAMs)](http://david.abcc.ncifcrf.gov/kegg.jsp?path=hsa04514$Cell%20adhesion%20molecules%20(CAMs)&termId=470038855&source=kegg) | 15 | 1.4 | 1.0E-2 | 2.1 |
| [Asthma](http://david.abcc.ncifcrf.gov/kegg.jsp?path=hsa05310$Asthma&termId=470038914&source=kegg) | 6 | 0.6 | 1.7E-2 | 3.9 |
| [O-Glycan biosynthesis](http://david.abcc.ncifcrf.gov/kegg.jsp?path=hsa00512$O-Glycan%20biosynthesis&termId=470038768&source=kegg) | 6 | 0.6 | 2.0E-2 | 3.7 |
| [Type I diabetes mellitus](http://david.abcc.ncifcrf.gov/kegg.jsp?path=hsa04940$Type%20I%20diabetes%20mellitus&termId=470038888&source=kegg) | 7 | 0.6 | 2.3E-2 | 3.1 |
| [Viral myocarditis](http://david.abcc.ncifcrf.gov/kegg.jsp?path=hsa05416$Viral%20myocarditis&termId=470038923&source=kegg) | 9 | 0.8 | 3.4E-2 | 2.4 |
| [Allograft rejection](http://david.abcc.ncifcrf.gov/kegg.jsp?path=hsa05330$Allograft%20rejection&termId=470038917&source=kegg) | 6 | 0.6 | 4.0E-2 | 3.1 |
| [Tight junction](http://david.abcc.ncifcrf.gov/kegg.jsp?path=hsa04530$Tight%20junction&termId=470038857&source=kegg) | 13 | 1.2 | 5.2E-2 | 1.8 |
| [Colorectal cancer](http://david.abcc.ncifcrf.gov/kegg.jsp?path=hsa05210$Colorectal%20cancer&termId=470038900&source=kegg) | 9 | 0.8 | 7.7E-2 | 2.0 |

KEGG_PATHWAY analysis of genes overexpressed in HeLa::REIIBP cells. The list was copiled using DAVID bionformatic resources (<http://david.abcc.ncifcrf.gov/>). Similar results were obtained using PANTHER and REACTOME pathways analysis. Fold enrichment measures the magnitude of enrichment compare to human genome. Fold enrichment > 1.5 was considered as interesting. The percentage is the total number of genes involved in a given term divided by the total number of input genes. P-values examine the significance of gene-term enrichment. P value <0.05 was considered significant. PANTHER and REACTOME_pathway analysis gave similar results.
