# Supplementary material for: A Novel Functional Role for MMSET in RNA Processing Based on the Link Between the REIIBP Isoform and Its Interaction with the SMN Complex
Source: PLoS One. 2014 Jun 12;9(6):e99493. doi: 10.1371/journal.pone.0099493 (PMC4055699; doi:10.1371/journal.pone.0099493)
Supplement: Table S4 — Polycomb regulated genes deregulated in HeLa::REIIBP cells. RNASeq result showing differently expressed transcripts which are known to be silenced by the Polycomb complex. (DOCX) [file pone.0099493.s008.docx]

**Table S4. Polycomb regulated genes deregulated in HeLa::REIIBP cells.**

| HeLa | HeLa::REIIBP | log2 fold change | Test stat | p value | q value | TSS Group |
| --- | --- | --- | --- | --- | --- | --- |
| 0.180989 | 209.443 | 10.1764 | 5.34154 | 5.00E-05 | 0.000699 | XIST |
| 0.023654 | 19.332 | 9.67471 | 5.67672 | 0.0026 | 0.020302 | hoxD13 |
| 0.092173 | 26.5154 | 8.16826 | 2.61464 | 5.00E-05 | 0.000699 | hoxD10,hoxD11 |
| 0.022791 | 5.67267 | 7.9594 | 0.536485 | 5.00E-05 | 0.000699 | ZFHX4 |
| 0.012297 | 0.799595 | 6.02294 | 0.449746 | 0.00065 | 0.006418 | TSHZ3 |
| 0.116205 | 5.24157 | 5.49526 | 1.26894 | 5.00E-05 | 0.000699 | hoxA-AS2 |
| 0.916566 | 27.5117 | 4.90766 | 2.9819 | 5.00E-05 | 0.000699 | hoxA2,hoxA3,hoxA4,hoxA5,hoxA6 |
| 0.155687 | 4.66897 | 4.90638 | 3.18072 | 5.00E-05 | 0.000699 | CDX2 |
| 0.335562 | 7.86494 | 4.55079 | 2.96846 | 5.00E-05 | 0.000699 | hoxA-AS3 |
| 0.146991 | 2.8141 | 4.25887 | 1.1049 | 5.00E-05 | 0.000699 | MNX1 |
| 0.122475 | 1.71582 | 3.80834 | 2.91384 | 3.00E-04 | 0.00334 | VAX2 |
| 0.076468 | 0.843141 | 3.46285 | 4.98411 | 5.00E-05 | 0.000699 | ALX4 |
| 0.225519 | 2.23561 | 3.30935 | 1.61549 | 5.00E-05 | 0.000699 | CERS4 |
| 6.67131 | 47.3314 | 2.82676 | 6.88281 | 5.00E-05 | 0.000699 | hoxA10,hoxA9,MIR196B,RP1-170O19.20 |
| 0.109423 | 0.704996 | 2.6877 | 0.895312 | 5.00E-05 | 0.000699 | NKX2-1,RP11-896J10.3,SFTA3 |
| 1.42929 | 8.71191 | 2.60769 | 5.95651 | 5.00E-05 | 0.000699 | MSX2 |
| 1.65491 | 9.77224 | 2.56194 | 3.20854 | 5.00E-05 | 0.000699 | SIX1 |
| 1.65491 | 9.77224 | 2.56194 | 3.20854 | 5.00E-05 | 0.000699 | SIX1 |
| 0.110248 | 0.637851 | 2.53246 | 1.88388 | 4.00E-04 | 0.004268 | EMX2 |
| 4.78754 | 26.4202 | 2.46428 | 2.98319 | 5.00E-05 | 0.000699 | hoxB3,hoxB4,hoxB5,hoxB6,hsa-mir-10a |
| 0.440164 | 2.32485 | 2.40102 | 4.84265 | 5.00E-05 | 0.000699 | POU3F2 |
| 0.340756 | 1.2974 | 1.92881 | 1.82296 | 5.00E-05 | 0.000699 | LHX6 |
| 0.860198 | 3.26568 | 1.92464 | 1.44372 | 0.002 | 0.016582 | hoxD3,hoxD4,MIR10B |
| 1.02304 | 3.78468 | 1.88731 | 2.59969 | 2.00E-04 | 0.002376 | hoxA13,RP1-170O19.14 |
| 2.37123 | 8.35358 | 1.81676 | 2.67148 | 5.00E-05 | 0.000699 | hoxA11-AS |
| 2.46125 | 7.88155 | 1.67909 | 3.53111 | 5.00E-05 | 0.000699 | SATB2 |
| 12.3909 | 34.6854 | 1.48505 | 3.28631 | 5.00E-05 | 0.000699 | hoxB7,hoxB8,hoxB9,MIR196A1,RP11-357H14.19,RP11-357H14.20 |
| 3.18897 | 7.0668 | 1.14797 | 1.71474 | 0.0041 | 0.029029 | hoxA11 |
| 2.18604 | 4.71268 | 1.10823 | 2.01499 | 8.00E-04 | 0.007628 | ISL2 |
| 3.34603 | 6.76344 | 1.01531 | 2.70139 | 0.00015 | 0.001852 | DLX2 |
| 6.99126 | 12.8142 | 0.874118 | 1.79249 | 0.0017 | 0.014516 | PBX3 |
| 6.99126 | 12.8142 | 0.874118 | 1.79249 | 0.0017 | 0.014516 | PBX3 |
| 1.22551 | 2.21261 | 0.852366 | 1.82265 | 0.0011 | 0.010081 | hoxB13 |
| 5.62595 | 9.81686 | 0.803164 | 1.96327 | 8.00E-04 | 0.007628 | NKX2-5 |
| 4.94395 | 8.25957 | 0.740402 | 1.84106 | 0.00155 | 0.01345 | MSX1 |
| 7.85477 | 4.7709 | -0.62509 | -1.87509 | 0.0018 | 0.015248 | AC090340.1,ONECUT2 |
| 37.7624 | 19.588 | -0.94698 | -2.22194 | 1.00E-04 | 0.001287 | RP11-838N2.4,TGIF1 |
| 3.61235 | 1.75277 | -1.0433 | -2.15479 | 7.00E-04 | 0.006802 | TLX3 |
| 8.54407 | 3.63146 | -1.23437 | -3.86521 | 5.00E-05 | 0.000699 | HMX3 |
| 1.01588 | 0.380619 | -1.41631 | -1.33941 | 6.00E-04 | 0.00598 | PAX8 |
| 9.76075 | 3.4515 | -1.49977 | -3.15511 | 5.00E-05 | 0.000699 | MKX |
| 12.863 | 3.95877 | -1.7001 | -2.64627 | 5.00E-05 | 0.000699 | PRRX2 |
| 0.882983 | 0.25344 | -1.80074 | -2.18719 | 5.00E-05 | 0.000699 | MEOX1 |

RNASeq result showing differently expressed transcripts which are known to be silenced by the Polycomb complex.
