# Supplementary material for: A Novel Functional Role for MMSET in RNA Processing Based on the Link Between the REIIBP Isoform and Its Interaction with the SMN Complex
Source: PLoS One. 2014 Jun 12;9(6):e99493. doi: 10.1371/journal.pone.0099493 (PMC4055699; doi:10.1371/journal.pone.0099493)
Supplement: Text S1 — Supplemental Experimental Procedures. (DOC) [file pone.0099493.s009.doc]

**Supplemental Experimental Procedures**

**Cell lines and viral transduction**

H929 and HeLa cell lines were grown in RPMI1640 containing GlutaMax™(Life Technology), and DMEM (Life Tecnology) respectively, both media were supplemented with 10% heat-inactivated fetal calf serum (Sigma). Cells were cultured at 37 °C in a humidiﬁed gas chamber with 95% air and 5% carbon dioxide. All cell lines used were mycoplasma-free as conﬁrmed by PCR. Cell identities were confirmed by short tandem repeats analysis. Proliferation assay, western blot, apoptosis and cell cycle analysis were described previously[^1-3^](#_ENREF_1). The lentivirus constructs were transduced into myeloma cells as described previously.

**Cloning**

cDNA for REIIBP was cloned in frame with the C-terminal double tags 6xHIS-3xFLAG and, through an auto-catalytic E2A peptide, linked to green fluorescence protein (GFP). The ΔSET constructs were generated by point mutating Asparagine 359, present in the SET domain, to Glycine. The construct was cloned into a lentivirus system (pRRLSIN) substituting the original GFP gene.

**Western Blotting**

Cells were harvested in RIPA buffer supplemented with Protease inhibitor cocktail (ROCHE). Cytoplasm and nuclear extracts were prepared by nuclei extraction method. Cells were resuspend in 10mM Hepes pH 7.9, 10mM KCl, 0.1 mM EDTA 0.1, mM EGTA 1x, Protease inhibitor cocktail (ROCHE) 1mM DTT, for 15 min on ice before adding 1% NP40 and vortexing for 10 seconds. Nuclei were then pelleted to separate them from the supernatant cytosolic fraction. Nuclei were lysed in RIPA buffer supplemented with 2% SDS and then sonicated. Protein concentration was estimated by BCA assay (Thermo Scientific). Proteins were resolved on 4-12% SDS–PAGE gradient gels (Novex, Life Tecnology), transferred to PVDF membrane (Millipore), blocked with either 5% milk or 5% BSA and incubated with primary antibody for 2 hours at room temperature or overnight at 4°C. Primary antibodies used were UNRIP, Histone3, 3MeK27H3 (Abcam), 2MeK36H3 (Active Motif), GEMIN3 (Novus Biologicals), SMN, GEMIN5 (Millipore), REIIBP/MMSETII (Abgene and [^6^](#_ENREF_6), FLAG (SIGMA). Membranes were then incubated with antimouse or antirabbit secondary antibodies conjugated to horseradish peroxidase (Amersham Biosciences) for 1 hour at room temperature. ECL-Plus (Amersham Biosciences) was used for protein band detection.

**Proliferation assays**

HeLa cell proliferation was measured by the colourimetric WST-1 assay (ROCHE). Cells were plated in 100ul of media at a density of 0.5x10^4^ cells per well in 96-well plates and incubated in a humidified atmosphere at 37^o^C with 5% CO_2_ for 4 days. To determine proliferation 10µl of WST-1 solution was then added to each well and the plates read on an Epoch plate reader (BIotec) after a further 30 min incubation.

**Sample digestion and mass spectrometry analysis**

Liquid Chromatography solvents were purchased from Rathburn (Walkerburn, UK). All other reagents were purchased from Sigma-Aldrich (Poole, UK) unless otherwise stated. 1D SDS-PAGE gel lanes corresponding to double affinity purification from H929::REIIBP and H929 were excised equivalently and in their entirety as 9 sections each. The gel sections were diced and reduced with 5 mM Tris(2-carboxyethyl)phosphine, free cysteines were alkylated with 55 mM iodoacetamide, and the proteins subjected to digestion with modified porcine Trypsin (Promega, Madison, W) overnight at 37°C in 50 mM triethylammonium bicarbonate. After digestion, the resulting peptides were extracted with acetonitrile and triethylammonium bicarbonate washes, the solution dried *in vacuo* and reconstituted in 0.1% formic acid for LC-MS/MS. Reversed phase chromatography was performed using an HP1200 platform (Agilent, Wokingham, UK). Forty per cent of each sample was analysed as a 4 µL injection. Peptides were resolved on a 75 µm I.D. 15 cm C18 packed emitter column (3 µm particle size; NIKKYO TECHNOS CO., LTD, Tokyo, Japan) over 30 min using a linear gradient of 96:4 to 50:50 buffer A:B (buffer A: 2% acetonitrile/0.1% formic acid; buffer B: 80% acetonitrile/0.1% formic acid) at 250 nL/min. Peptides were ionised by electrospray ionisation using 1.7 kV applied immediately pre-column to the packed emitter via a microtee built into the nanospray source. The sample was infused into an LTQ Velos Orbitrap mass spectrometer directly from the end of the tapered tip silica column (6-8 µm tapered tip). The ion transfer tube was heated to 200°C and the S-lens set to 60%. MS/MS were acquired using data dependent acquisition based on a full 30,000 resolution FT-MS scan to sequence the top 10 most intense ions using enhanced ion trap scans and collision-induced dissociation. Automatic gain control was set to 1,000,000 for FT-MS and 30,000 for IT-MS/MS, full FT-MS maximum inject time was 500 ms and normalised collision energy was set to 35% with an activation time of 10 ms. Wideband activation was used to co-fragment precursor ions undergoing neutral loss of up to -20 m/z from the parent ion, including loss of water/ammonia. MS/MS was acquired for selected precursor ions with a single repeat count followed by dynamic exclusion with a 10 ppm mass window for 15 s duration based on a maximal exclusion list of 500 entries. Raw MS/MS data were compiled into peaklists using Proteome Discoverer v1.2 (Thermo Fisher Scientific, Hemel Hempstead, UK) default parameters. Peaklists were interrogated against a SwissProt 2011_01 *homo sapiens* subset database (20,282 sequences) customised to include the REIIBP construct sequence using Mascot v2.2 ([www.matrixscience.com](http://www.matrixscience.com/)) and assuming tryptic enzyme specificity with up to two missed cleavages. A precursor ion tolerance of 5 ppm and fragment ion tolerance of 0.25 Da was applied and the following variable modifications were accounted for: acetylation of the protein N-terminus, carbamidomethylation of cysteine, oxidation of methionine, pyroglutamisation of peptide N-terminal glutamine and phosphorylation of serine, threonine and tyrosine. The Mascot peptide and protein identification results were grouped and validated using Scaffold v3.0 (Proteome Software Inc., Portland, OR). Protein identifications were automatically accepted if they contained at least 2 unique peptides assigned with at least 95% confidence by Peptide Prophet [^7^](#_ENREF_7) Relative protein enrichment was inferred from the spectral counts observed for each protein between the control and H929::REIIBP samples [^8^](#_ENREF_8). For the purpose of assessing enrichment, the protein acceptance probability was decreased to 80%. Spectra at this lower threshold were confirmed to be of good quality by visual inspection, and no false discovery hits against a reversed sequence decoy database were reported at this confidence level.

**RNA extraction and qRT-PCR**

All RNA was purified by Trizol method (Life Tecnology) and DNase treated (Zymo Research). Reverse transcription was conducted using Monster script (CAMBIO EPIGENOME), following manual recommendations. 6mers oligos were used for priming RNA except in the intron retention experiment, where specific priming oligos were used. All qPCR was done using syber green and a relative standard curve method. Absence for DNA contamination was carried by analysing control samples without Reverse transcriptase.

**List of primers used for qRT-**PCR

| **U1 snRNA** | GATACCATGATCACGAAGGTGGTT CACAAATTATGCAGTCGAGTTTCC |
| --- | --- |
| **U2 snRNA** | TTTGGCTAAGATCAAGTGTAGTATCTGTTC AATCCATTTAATATATTGTCCTCGGATAGA |
| **U4 snRNA** | GCGCGATTATTGCTAATTGAAA AAAAATTGCCAATGCCGACTA |
| **U5 snRNA** | GGTTTCTCTTCAGATCGCATAAATC CTCAAAAAATTGGGTTAAGACTCAGA |
| **U12 snRNA** | AACTTATGAGTAAGGAAAATAACGATTCG CGACCTTTACCCGCTCAAAA |
| **βactin** | CCCTGGCACCCAGCAC GCCGATCCACACGGAGTAC |
| **AC015987.1 exon** | CTCCAAGAAATGTGGAGCAA GTAGCCAGCACATCCCAGAT |
| **AC015987.1 intron** | TTTGAGTCAATCAAGCTTCTGG CAGCTAATGTGTCAGCAGCA |
| **RP6-24A23.6 intron** | ATCCTTGAGCCCAGGAGTCT GAGGTCTCACTATGCTGCCC |
| **RP6-24A23.6 exon** | GCCTACAAGTGGACTCTGGC TCAAAGGGACAGACTGGGGA |
| **TMOD3 exon** | AACCCACATCCTTGCTTTGT ACTCGGTCAACTGGAAAACA |
| **TMOD3 intron** | CCATGTCACCCAAGAGAAGCA ACCCCAAGAACTCATAGCCC |
| **SCO2 exon** | CCAGTGCAGCCTGTCTTCAT ACGCGGTAACTGTGACTAGC |
| **SCO2 intron** | GCTGGTGATCCCAGGTAGAG TCAGGCTTAACAGGCATTTG |
| **VMP1 intron** | CTTATGGACCTTTGCTGCCC AGTCGCCATGCAGATACTCA |
| **VMP1 exon** | AGCAGCAAACTTGCAACAGA GTGCGGTGGGAATTCTCTAA |

**RNA seq**

Total RNA was purified using the Trizol (life Technology) method. Total RNA from 3 replicates of HeLa cell line transduced with cDNA coding for REIIBP and 3 replicates of the control cell line (HeLa) was extracted. 2 µg total RNA from each sample was used. rRNA was depleted by using Ribozero magnetic kit (Epicentre), with post-depletion clean-up. The library was prepared using NEBNext mRNA Library Prep (New England Biolabs) with the following modifications: After fragmentation, Ampure XP clean-up (2.8x volume ratio), 2 times 80% ethanol washes, 14.5 µl EB. Reverse transcription (random primers) with Superscript II. Post-reverse transcription clean-up with Ampure XP (1.2x volume ratio), 2 times 80% Ethanol washes, 40 µl EB. 50ul reaction for end repair (40 µl cDNA, 5 µl reaction buffer, 5 µl enzyme mix). Post end repair, A-tailing and adapter ligation clean-ups with Ampure XP as in protocol. Custom adapters were used at ligation. There was no size selection. Library was amplified by PCR for 12 cycles. The six samples were run over two lanes HiSeq 2000, with a standard 100 cycle run. Raw fastq files were aligned using Tophat 2.0.8 (http://tophat.cbcb.umd.edu/) with default parameters, and Cufflinks 2.1.1 (http://cufflinks.cbcb.umd.edu/) was used to estimate transcript abundance and calculate differential expression between REIIBP and control samples. The results were visualized using cummerbund 2.0.0 (<http://compbio.mit.edu/cummeRbund/>).

**Statistics**

Statistical analysis was performed using a two-tailed student t-test. P-value < 0.05 was considered statistically signiﬁcant.

**Supplemental References**

1. Aronson LI, Davenport EL, Mirabella F, Morgan GJ, Davies FE. Understanding the interplay between the proteasome pathway and autophagy in response to dual PI3K/mTOR inhibition in myeloma cells is essential for their effective clinical application. *Leukemia*. 2013.

2. Davenport EL, Moore HE, Dunlop AS, et al. Heat shock protein inhibition is associated with activation of the unfolded protein response pathway in myeloma plasma cells. *Blood*. 2007;110(7):2641-2649.

3. Zhang L, Fok JJ, Mirabella F, et al. Hsp70 inhibition induces myeloma cell death via the intracellular accumulation of immunoglobulin and the generation of proteotoxic stress. *Cancer Lett*. 2013;339(1):49-59.

4. Wiznerowicz M, Trono D. Conditional suppression of cellular genes: lentivirus vector-mediated drug-inducible RNA interference. *J Virol*. 2003;77(16):8957-8961.

5. Zufferey R, Nagy D, Mandel RJ, Naldini L, Trono D. Multiply attenuated lentiviral vector achieves efficient gene delivery in vivo. *Nat Biotechnol*. 1997;15(9):871-875.

6. Brito JL, Walker B, Jenner M, et al. MMSET deregulation affects cell cycle progression and adhesion regulons in t(4;14) myeloma plasma cells. *Haematologica*. 2009;94(1):78-86.

7. Keller A, Nesvizhskii AI, Kolker E, Aebersold R. Empirical statistical model to estimate the accuracy of peptide identifications made by MS/MS and database search. *Anal Chem*. 2002;74(20):5383-5392.

8. Liu H, Sadygov RG, Yates JR, 3rd. A model for random sampling and estimation of relative protein abundance in shotgun proteomics. *Anal Chem*. 2004;76(14):4193-4201.
